# The origin of the medial circumflex femoral artery: a meta-analysis and proposal of a new classification system

**DOI:** 10.7717/peerj.1726

**Published:** 2016-02-29

**Authors:** Krzysztof A. Tomaszewski, Brandon M. Henry, Jens Vikse, Joyeeta Roy, Przemysław A. Pękala, Maren Svensen, Daniel L. Guay, Karolina Saganiak, Jerzy A. Walocha

**Affiliations:** 1 Department of Anatomy, Jagiellonian University Medical College, Kraków, Poland; 2 International Evidence-Based Anatomy Working Group, Kraków, Poland

**Keywords:** Medial circumflex femoral artery, Meta-analysis, Evidence-based anatomy, Common femoral artery, Deep femoral artery

## Abstract

**Background and Objectives.** The medial circumflex femoral artery (MCFA) is a common branch of the deep femoral artery (DFA) responsible for supplying the femoral head and the greater trochanteric fossa. The prevalence rates of MCFA origin, its branching patterns and its distance to the mid-inguinal point (MIP) vary significantly throughout the literature. The aim of this study was to determine the true prevalence of these characteristics and to study their associated anatomical and clinical relevance.

**Methods.** A search of the major electronic databases Pubmed, EMBASE, Scopus, ScienceDirect, Web of Science, SciELO, BIOSIS, and CNKI was performed to identify all articles reporting data on the origin of the MCFA, its branching patterns and its distance to the MIP. No data or language restriction was set. Additionally, an extensive search of the references of all relevant articles was performed. All data on origin, branching and distance to MIP was extracted and pooled into a meta-analysis using MetaXL v2.0.

**Results.** A total of 38 (36 cadaveric and 2 imaging) studies (*n* = 4,351 lower limbs) were included into the meta-analysis. The pooled prevalence of the MCFA originating from the DFA was 64.6% (95% CI [58.0–71.5]), while the pooled prevalence of the MCFA originating from the CFA was 32.2% (95% CI [25.9–39.1]). The CFA-derived MCFA was found to originate as a single branch in 81.1% (95% CI [70.1–91.7]) of cases with a mean pooled distance of 50.14 mm (95% CI [42.50–57.78]) from the MIP.

**Conclusion.** The MCFA’s variability must be taken into account by surgeons, especially during orthopedic interventions in the region of the hip to prevent iatrogenic injury to the circulation of the femoral head. Based on our analysis, we present a new proposed classification system for origin of the MCFA.

## Introduction

The medial circumflex femoral artery (MCFA) is a posteromedial branch of the deep femoral artery (DFA), also called the profunda femoris, or less frequently, the common femoral artery (CFA) (Mamatha et al., 2012). The MCFA branches into numerous divisions, its first perforating the hip capsule and continuing as the inferior retinacular artery to supply the femoral head and the greater trochanteric fossa. The deep branch of the MCFA courses the obturator externus and along the conjoint tendon before perforating the femoral head capsule (Li & Cole, 2015). This intracapsular branch courses the posterior aspect of the femoral neck before it ends beneath the synovium as the superior retinacular branches (Gautier et al., 2000). The two retinacular branches of the MCFA are closely associated with the femoral neck, putting them at risk during femoral neck fractures. Boraiah et al. (2009) reported the prevalence of avascular necrosis of the femoral head post-fracture ranging from 10 to 30% when utilizing internal fixation as the surgical technique.

Embryologically, the lower limb buds arise from the lateral aspect of the L2-S2 segments of the trunk during the 5th week of gestation (Moore, Agur & Dalley, 2014). During development, the primary axial artery is vital for lower limb vascular supply and differentiation. The femoral vasculature develops as the sciatic artery regresses (Kalhor et al., 2009), but the MCFA itself develops independently from the rete femorale (Perera, 1995). Increased blood flow through the rete femorale capillaries during organogenesis is a generally accepted mechanism determining the mature arterial branching pattern (Sahin et al., 2003).

Clinically, the anatomy of the MCFA is relevant in decreasing the incidence of avascular necrosis of the femoral head during embolization and procedures such as arterial catheterization and hip surgery (Kalhor et al., 2009; Oide, 1979). In orthopedic surgeries of the hip region, the Kocher–Langenbeck (KL) approach is an important technique allowing for optimum exposure to certain acetabular fractures, most notably those affecting the posterior component of the acetabulum. Without proper anatomical information on this region there is a substantial risk of vascular injury to the MCFA (Freitas et al., 2012).

Significant differences have been reported in the arterial origins of the MCFA, for example that the MCFA originates from the DFA in 20% (Emura et al., 1989) to 86% (Massoud & Fletcher, 1997) of individuals, and from the CFA in 5% (Siddharth et al., 1985) to 80% (Emura et al., 1989) of subjects. Other rarer variations of the MCFA origin, including from the superficial femoral artery (SFA) (Siddharth et al., 1985), the external iliac artery (Clarke & Colborn, 1993), and even the LCFA (Nasr et al., 2014) have also been reported in the literature. Knowledge of the origin of the MCFA is essential in orthopedics as a means to avoid iatrogenic vascular necrosis of the femoral head in procedures such as trochanteric and intertrochanteric osteotomies (Gautier et al., 2000; Manjappa & Prasanna, 2014).

Due to the significant reported variability in the origin of the MCFA and its high level of clinical significance, the aim of our study was to determine the population prevalence estimates of the MCFA branching localizations and to study their associated anatomical characteristics. Additionally, we aimed to establish a new universal classification system for variations in the origin of the MCFA.

## Materials and Methods

### Search strategy

An extensive search of the major electronic databases (Pubmed, EMBASE, Scopus, ScienceDirect, Web of Science, SciELO, BIOSIS, and CNKI) was conducted through July 2015 in order to identify articles eligible for inclusion into the meta-analysis. To identify all potentially relevant anatomical data, a comprehensive search of all articles related to femoral circulation was performed. The following search terms were used: femoral head circulation, femoral head blood supply, femoral neck circulation, femoral neck blood supply, superior gluteal artery, inferior gluteal artery, medial femoral circumflex artery, lateral femoral circumflex artery, superficial femoral artery, deep femoral artery, retinacular arteries, extracapsular arterial ring of femoral neck, intracapsular arterial ring of femoral neck, arteries of the round ligament, posterior superior nutrient artery, posterior inferior nutrient artery, piriformis branch of the IGA, and profunda femoris. The authors did not set any date or language restrictions. Additionally, a broad search of the references of all relevant manuscripts was conducted to identify further eligible articles. Authors of the study strictly followed the Preferred Reporting Items for Systematic Reviews and Meta-analyses (PRISMA) guidelines (Supplemental Information 1).

### Eligibility assessment

Eligibility for inclusion into the study was assessed by two reviewers (JV and MS). All studies reporting extractable data on the anatomy of the MCFA were included into the meta-analysis. The exclusion criteria for the meta-analysis included: (1) reviews, case reports, letters to the editor, conference abstracts, (2) reporting incomplete or non-extractable data, (3) studies on patients with congenital hip or femur pathologies, and (4) animal studies. All studies written in languages not spoken fluently by any of the authors, were translated by medical professionals, who are fluent in the original language of the publication and English. In the case of any inconsistencies or disagreements during the study selection process, all decisions were made by a consensus between all the reviewers, after consulting with the authors of the original study if possible.

### Data extraction

Two reviewers (JV and MS) independently extracted all the eligible data from the studies included in the meta-analysis. Data on the sample size, year of study, type of study, geographical region, gender, side, prevalence of the various origins of the MCFA, and the mean distance of the MCFA originating from the DFA and the CFA to the mid-inguinal point (MIP) were extracted. As only some studies reported specific data on the different types of CFA origins of the MCFA and the different types of DFA origins of the MCFA, we extracted them separately for the purposes of analysis.

Authors of all articles containing inconsistent data were contacted by email for additional information when possible. Additionally, authors excluded from the analysis morphometric data (mean length) from studies on fetuses, due to the lack of comparability of such data with that obtained from adult samples.

### Statistical analysis

Statistical analysis was performed by BMH, JV, and JR using MetaXL version 2.0 by EpiGear International Pty Ltd (Wilston, Queensland, Australia) to calculate multi-categorical pooled prevalence estimates for the various origins of the MCFA (Henry et al., 2015). The morphometric data was pooled into an analysis using Comprehensive Meta-Analysis version 3.0 by Biostat (Englewood, New Jersey, USA). All analyses were performed by using a random effects model.

The Chi^2^ test and Higgins *I*^2^ statistic were used to measure heterogeneity among the studies included in the meta-analysis. A Cochran’s Q *p*-value of <0.10 for the Chi^2^ test was regarded as indicator of significant heterogeneity between studies (Higgins & Green, 2011). For Higgins *I*^2^, values of 0–40% were considered as “might not be important”; 30–60% “might indicate moderate heterogeneity”; 50–90% “may indicate substantial heterogeneity”; and 75–100% “may represent considerable heterogeneity” (Higgins & Green, 2011).

To probe the sources of heterogeneity, subgroup analysis by type of study, geographical distribution, gender, and side (left vs. right) and/or a sensitivity analysis inclusive of studies with a number of lower limbs ≥100, was conducted. Confidence intervals were used to determine statistically significant differences between 2 or more groups. In the case of overlapping confidence intervals, the differences were regarded as statistically insignificant (Henry et al., 2015).

### Establishment of a classification system

In order to establish a universally applicable classification system for the origin of MCFA, the authors set an a priori threshold level of a minimum 1% pooled prevalence of a variant in the overall analysis for it to be eligible for inclusion in the classification system. For any sub-variants not represented in the overall analysis, eligibility for inclusion was determined by multiplying the pooled prevalence of the particular sub-variant by the pooled prevalence of its main variant representative in the overall analysis. If the calculated value was ≥1%, it would be deemed eligible for inclusion as independent variant in the new classification. All variants below a pooled prevalence of ≤1% were considered as anomalies. For an anomaly to be included in the classification system, a threshold of ≥0.5% pooled prevalence in the overall analysis was set.

**Figure 1:**
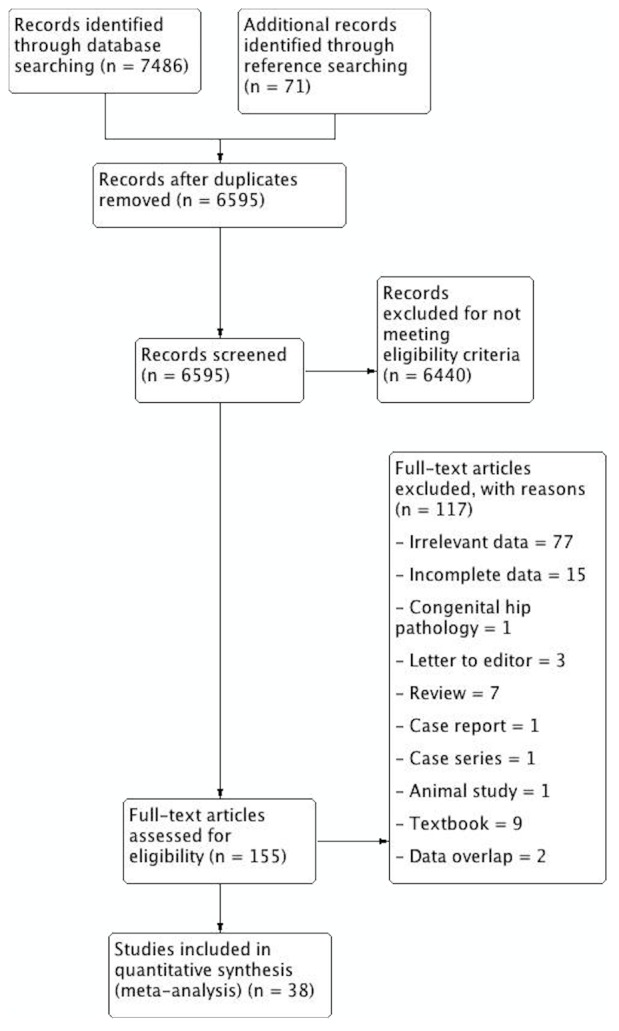
PRISMA flowchart of study identification, evaluation and inclusion into the meta-analysis.

## Results

### Study identification

The study identification process is summarized in Fig. 1. Major databases were extensively searched to initially identify 7,486 articles. A further 71 articles were added by searching the references of included articles. A total of 155 articles were assessed by full text for potential eligibility. One hundred and twenty-five articles were deemed ineligible for inclusion, and 38 articles were finally included into the meta-analysis.

### Characteristics of included studies

The characteristics of included studies are summarized in Table 1. A total of 38 studies (*n* = 4,351 lower limbs) were included in the meta-analysis. The studies spanned from 1934 to 2015 and demonstrated a wide geographical distribution with studies hailing from North America, Europe, Asia and Africa. The type of study included in the meta-analysis was predominantly cadaveric, except for studies by Massoud & Fletcher (1997) and Gościcka, Gielecki & Zietek (1990) who used digital subtraction transfemoral aortogram and radiograms, respectively.

**Table 1:** Characteristics of included studies.

Study	Country	Type of study	*n* (# of lower limbs)
Al-Talalwah (2015)	Scotland	Cadaveric	342
Darji et al. (2015)	India	Cadaveric	130
Manjappa & Prasanna (2014)	India	Cadaveric	40
Nasr et al. (2014)	Saudi Arabia	Cadaveric	90
Anwer, Karmalkar & Humbarwadi (2013)	India	Cadaveric	60
Lalovic et al. (2013)	Serbia	Cadaveric	42
Peera & Sugavasi (2013)	India	Cadaveric	40
Shiny Vinila et al. (2013)	India	Cadaveric	40
Zlotorowicz et al. (2013)	Poland	Cadaveric	16
Kalhor et al. (2012)	Iran	Cadaveric	35
Del Sol, Galdames & Vasquez (2011)	Chile	Cadaveric	92
Dixit et al. (2011)	India	Cadaveric	228
Prakash et al. (2010)	India	Cadaveric	64
Boraiah et al. (2009)	USA	Cadaveric	14
Vasquez et al. (2007)	England	Cadaveric	438
Tanyeli et al. (2006)	Turkey	Cadaveric	100
Dixit, Mehta & Kothari (2001)	India	Cadaveric	48
Liu, Huang & Zhao (2001)	China	Cadaveric	50
Gautier et al. (2000)	Switzerland	Cadaveric	24
Munteaunu, Burcoveanu & Andriescu (1998)	Romania	Cadaveric	50
Luo et al. (1997)	China	Cadaveric	50
Massoud & Fletcher (1997)	USA	Imaging (Digital Subtraction Transfemoral Aortogram)	188
Clarke & Colborn (1993)	USA	Cadaveric	30
Gościcka, Gielecki & Zietek (1990)	Poland	Imaging (Radiogram)	100
Emura et al. (1989)	Japan	Cadaveric	337
Boonkham & Plakornkul (1987)	Thailand	Cadaveric	113
Siddharth et al. (1985)	USA	Cadaveric	100
O’Hara & Dommisse (1983)	South Africa	Cadaveric	19
Bloda, Sierociński & Kling (1982)	Poland	Cadaveric	80
Marcade et al. (1978)	France	Cadaveric	50
Chung (1976)	USA	Cadaveric	20
Ogden (1974)	USA	Cadaveric	36
Gremigni (1968)	Italy	Cadaveric	100
De Beer (1965)	South Africa	Cadaveric	180
Keen (1961)	South Africa	Cadaveric	280
Ming-Tzu (1937)	China	Cadaveric	150
Williams, Martin & McIntire (1934)	USA	Cadaveric	481
Lipshutz (1916)	USA	Cadaveric	95

### Origins of the medial circumflex femoral artery

**Figure 2:**
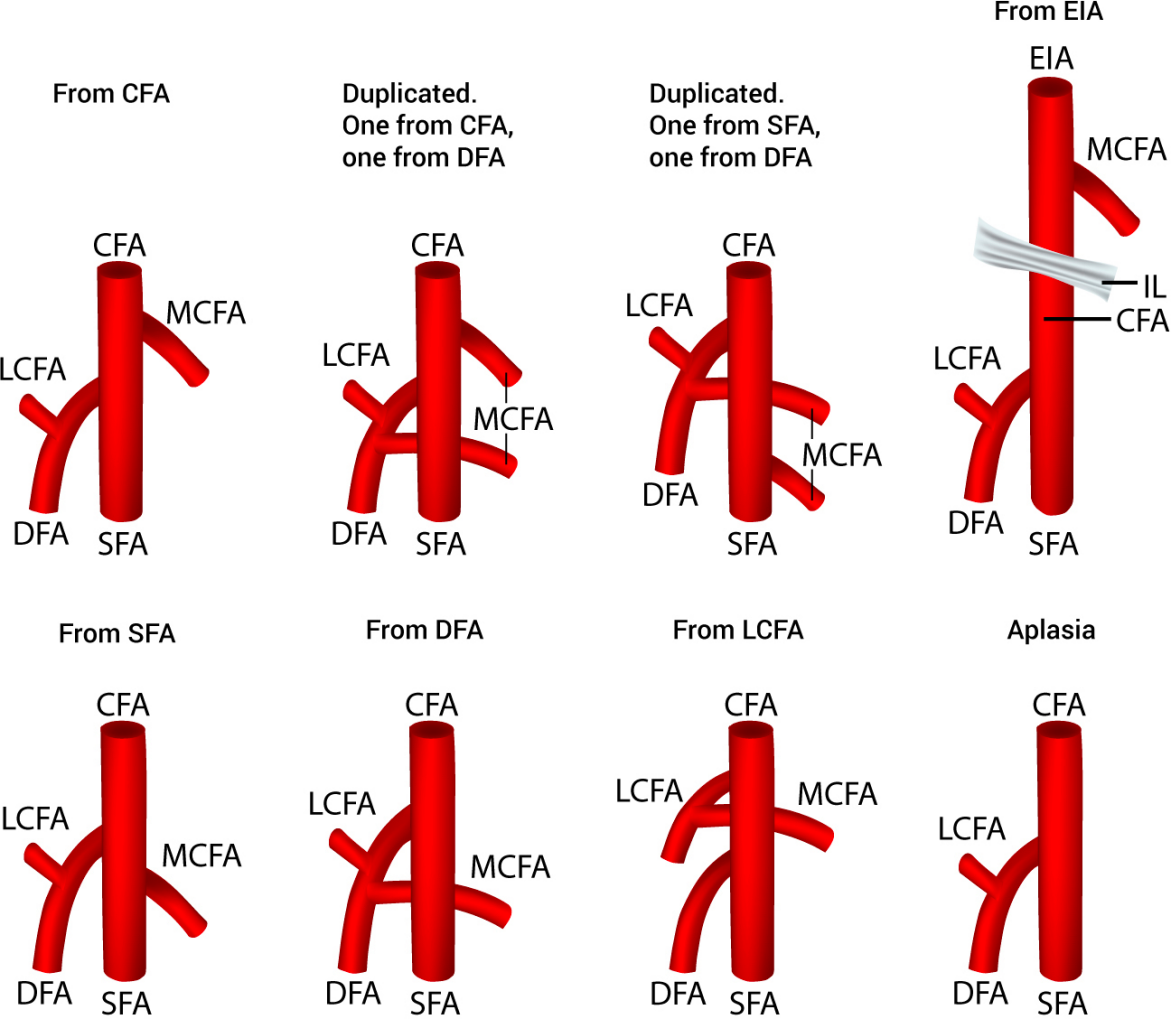
Variants of the medial circumflex femoral artery origins. CFA, common femoral artery; DFA, deep femoral artery; EIA, external iliac artery; IL, inguinal ligament; LCFA, lateral circumflex femoral artery; MCFA, medial circumflex femoral artery; SFA, superficial femoral artery.

**Figure 3:**
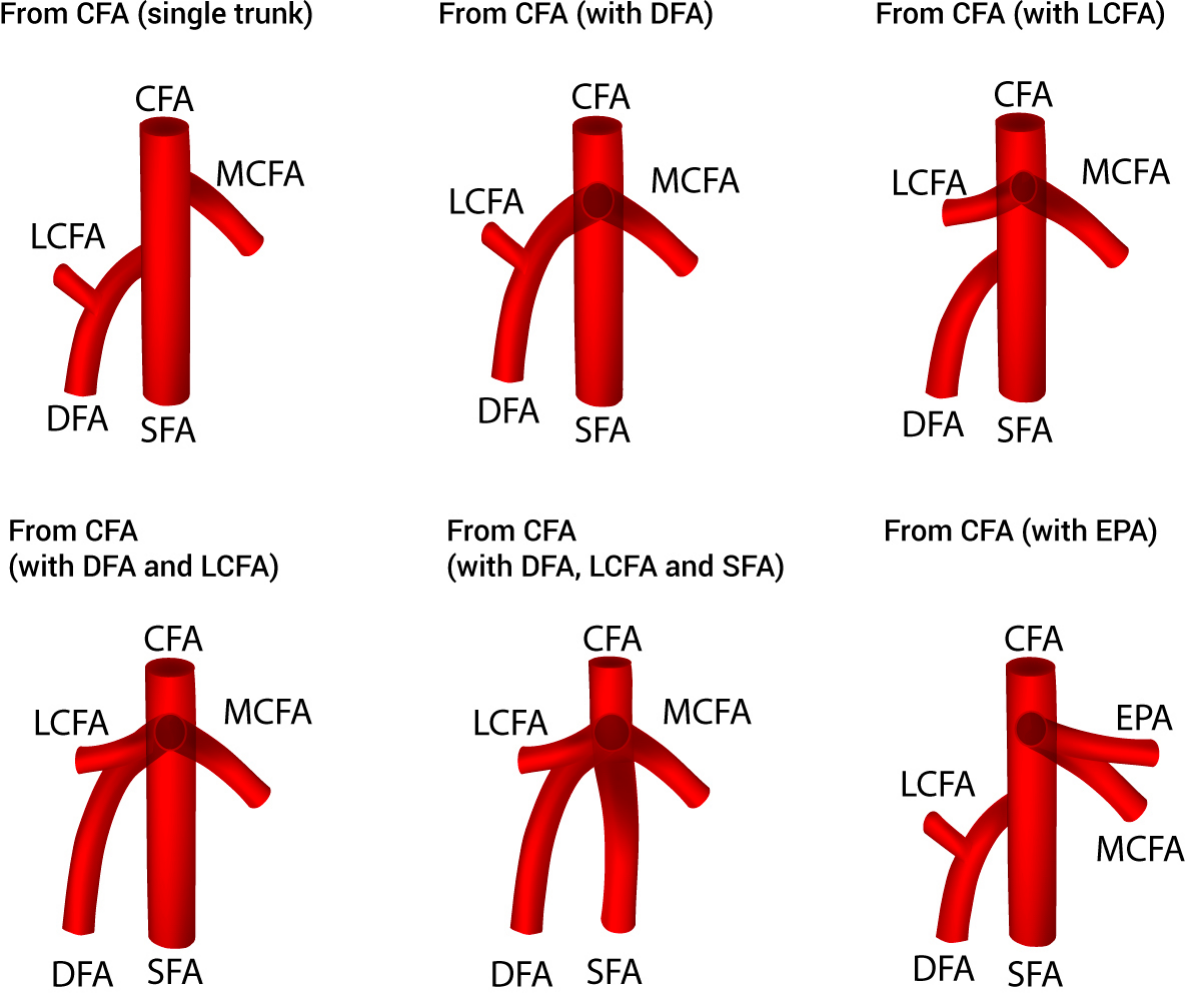
Sub-variants of the medial circumflex femoral artery origins from the common femoral artery. CFA, common femoral artery; DFA, deep femoral artery; EPA, external pudendal artery; LCFA, lateral circumflex femoral artery; MCFA, medial circumflex femoral artery; SFA, superficial femoral artery.

Thirty-eight studies (*n* = 4,351 lower limbs) reported data on the prevalence of the various origins of the MCFA. After a thorough review of the literature, we identified eight different variants of MCFA origins (Fig. 2), with 6 different sub-variants of MCFA origins from the CFA (Fig. 3). The pooled results are reported in Table 2 (Supplemental Information 2). Our analysis demonstrated that the MCFA most commonly originates from the DFA, with a pooled prevalence of 64.6% (95% CI [58.0–71.5]). The second most common origin of the MCFA was from the CFA with a pooled prevalence of 32.2% (95% CI [25.9–39.1]), of which it originates as a single branch in 81.1% (95% CI [70.1–91.7]) of these cases (Table 3).

**Table 2:** Prevalence of the various origins of the MCFA with subgroup and sensitivity analyses.

Population	All	Africa	Asia	Europe	North America	Cadaveric	Imaging	Sensitivity analysis (*n* ≥ 100 limbs)
Number of studies (number of legs)	38 (4,351)	3 (479)	15 (1,484)	10 (1,242)	8 (964)	34 (3,963)	2 (238)	15 (3,267)
From CFA: % (95% CI)	32.2 (25.9–39.1)	37.9 (33.9–42.6)	30.9 (19.0–45.3)	32.8 (25.2–41.9)	28.9 (10.8–49.9)	33.2 (26.6–40.5)	23.6 (0–71.7)	27.8 (17.6–37.8)
Duplicated. One from CFA, one from DFA: % (95% CI)	0.4 (0–1.6)	0.1 (0–0.6)	0.5 (0–3.5)	0.3 (0–1.8)	0.5 (0–5.9)	0.4 (0–1.7)	0.3 (0–19.6)	0.2 (0–2.0)
Duplicated. One from SFA, one from DFA: % (95% CI)	0.4 (0–1.5)	0.1 (0–0.6)	0.3 (0–3.1)	0.3 (0–1.8)	0.6 (0–6.4)	0.4 (0–1.6)	0.3 (0–19.6)	0.2 (0–1.8)
From SFA: % (95% CI)	1.0 (0–3.0)	0.1 (0–0.6)	0.6 (0–3.7)	1.1 (0–3.4)	3.5 (0–13.4)	1.0 (0–3.1)	2.8 (0–31.9)	1.4 (0–4.6)
From DFA: % (95% CI)	64.6 (58.0–71.5)	61.3 (57.4–66.1)	66.7 (53.9–80.3)	63.8 (56.0–72.9)	64.5 (41.5–83.1)	63.6 (56.8–70.9)	70.7 (20.1–100)	69.9 (57.4–78.5)
From LCFA: % (95% CI)	0.4 (0–1.6)	0.1 (0–0.6)	0.3 (0–3.1)	0.4 (0–2.0)	0.5 (0–5.9)	0.4 (0–1.7)	0.3 (0–19.6)	0.2 (0–1.8)
From EIA: % (95% CI)	0.4 (0–1.6)	0.1 (0–0.6)	0.3 (0–3.1)	0.3 (0–1.8)	0.7 (0–6.8)	0.4 (0–1.7)	0.3 (0–19.6)	0.1 (0–1.7)
Aplasia: % (95% CI)	0.5 (0–1.8)	0.1 (0–0.6)	0.3 (0–3.1)	0.8 (0–2.8)	0.8 (0–7.0)	0.5 (0–1.8)	1.7 (0–27.7)	0.3 (0–2.1)
*I*^2^: % (95% CI)	95.22 (94.20–96.06)	0 (0–83.95)	96.49 (95.33–97.36)	87.24 (78.52–92.41)	97.10 (95.77–98.01)	95.12 (94.00–96.03)	97.23 (92.91–98.91)	97.62 (96.93–98.15)
Cochran’s q, *p*-value	<0.001	0.523	<0.001	<0.001	<0.001	<0.001	<0.001	<0.001

**Notes.**

CFA common femoral artery DFA deep femoral artery EIA external iliac artery LCFA lateral circumflex femoral artery MCFA medial circumflex femoral artery SFA superficial femoral artery

**Table 3:** Prevalence of the various types of CFA origins of the MCFA with subgroups and sensitivity analyses.

Population	All	Africa	Asia	Europe	North America	Cadaveric	Imaging	Sensitivity analysis (*n* ≥ 100 limbs)
Number of studies (number of legs)	30 (1,416)	3 (183)	11 (519)	10 (422)	5 (242)	28 (1,367)	2 (36)	5 (753)
From CFA (single trunk): % (95% CI)	81.1 (70.1–91.7)	97.1 (95.5–100)	70.2 (47.6–92.9)	77.7 (54.1–94.3)	93.9 (90.7–99.9)	82.3 (71.8–93.0)	88.6 (71.9–100)	90.3 (54.7–100)
From CFA (with DFA): % (95% CI)	12.2 (4.4–23.2)	0.4 (0–2.4)	25.5 (6.9–52.0)	9.9 (0–29.2)	1.2 (0–4.6)	11.1 (3.6–22.0)	1.3 (0–15.1)	2.9 (0–17.2)
From CFA (with LCFA): % (95% CI)	1.7 (0–6.3)	1.4 (0–4.5)	1.0 (0–9.5)	1.9 (0–11.0)	2.9 (0–7.8)	1.5 (0–6.0)	6.1 (0–28.1)	1.8 (0–14.3)
From CFA (with DFA and LCFA): % (95% CI)	2.5 (0–7.8)	0.4 (0–2.4)	1.3 (0–10.3)	6.8 (0–20.9)	0.7 (0–3.5)	2.6 (0–8.1)	1.3 (0–15.1)	2.6 (0–16.5)
From CFA (with DFA, LCFA and SFA): % (95% CI)	1.4 (0–5.6)	0.4 (0–2.4)	1.0 (0–9.5)	2.4 (0–12.2)	0.7 (0-3.5)	1.4 (0–5.8)	1.3 (0–15.1)	1.9 (0–14.6)
From CFA (with EPA): % (95% CI)	1.1 (0–5.0)	0.4 (0–2.4)	1.0 (0–9.5)	1.3 (0–9.4)	0.7 (0–3.5)	1.1 (0–5.1)	1.3 (0–15.1)	0.4 (0–9.1)
*I*^2^: % (95% CI)	96.12 (95.23–96.83)	42.38 (0–82.59)	95.99 (94.31–97.18)	95.02 (92.61–96.64)	56.28 (0–83.81)	96.08 (95.15–96.83)	73.49 (0–94.02)	98.48 (97.73–98.99)
Cochran’s q, *p*-value	<0.001	0.176	<0.001	<0.001	0.057	<0.001	0.052	<0.001

**Notes.**

CFA common femoral artery DFA deep femoral artery EPA external pudendal artery LCFA lateral circumflex femoral artery MCFA medial circumflex femoral artery SFA superficial femoral artery

To probe the sources of heterogeneity, a sensitivity analysis was performed on 15 studies (*n* = 3,267 lower limbs) by limiting inclusion to studies with a sample size of 100 or more lower limbs (Table 2). Our results showed no significant deviations from the results of the overall analysis. Furthermore, subgroup analysis according to the geographical region was also performed (Table 2). Our results showed that the results were mostly consistent with our overall analysis, with all population subgroups demonstrating the MCFA originating from the DFA as the most common pattern of origin.

### Origins of the medial circumflex femoral artery with respect to gender and side

Five studies (*n* = 894 lower limbs; 683 male and 211 female) reported the prevalence of the various origins of MCFA according to gender (Table 4). Our results showed that the prevalences of the various origins of MCFA are comparable in both sexes and consistent with our overall result. When the MCFA originated from the CFA, it usually originated as a single trunk in both males and females. This was consistent with the results of our overall analysis. Further results on the origins of the MCFA with respect to gender are presented in Tables 4 and 5.

**Table 4:** Prevalence of the various origins of the MCFA with respect to gender.

	Male			Female		
	Total	Right	Left	Total	Right	Left
Number of studies (number of legs)	5 (683)	4 (268)	4 (269)	5 (211)	4 (88)	4 (89)
From CFA: % (95% CI)	32.4 (21.7–44.0)	29.4 (16.4–45.2)	28.7 (14.5–45.9)	32.7 (23.2–44.2)	32.3 (21.1–46.3)	30.9 (19.8–44.7)
From SFA: % (95% CI)	1.5 (0–5.0)	2.7 (0–9.0)	1.8 (0–7.8)	1.8 (0–5.4)	2.7 (0–8.0)	2.7 (0–7.9)
From DFA: % (95% CI)	64.9 (53.0–75.7)	66.0 (51.4–81.0)	66.8 (50.3–82.6)	64.2 (54.3–75.5)	62.9 (51.1–76.8)	64.3 (52.7–78.1)
From LCFA: % (95% CI)	0.3 (0–2.4)	0.6 (0–4.4)	0.7 (0–5.0)	0.6 (0–3.0)	1.1 (0–4.8)	1.1 (0–4.8)
From EIA: % (95% CI)	0.6 (0–3.2)	0.6 (0–4.4)	1.4 (0–7.0)	–	–	–
Aplasia: % (95% CI)	0.3 (0–2.4)	0.6 (0–4.4)	0.7 (0–5.0)	0.6 (0–3.0)	1.1 (0–4.8)	1.1 (0–4.8)
*I*^2^: % (95% CI)	85.5 (68.01–93.43)	72.35 (21.77–90.23)	77.12 (37.67–91.60)	58.38 (0–84.51)	32.02 (0–75.72)	32.42 (0–75.94)
Cochran’s q, *p*-value	<0.001	0.013	0.004	0.048	0.220	0.218

**Notes.**

CFA common femoral artery DFA deep femoral artery EIA external iliac artery LCFA lateral circumflex femoral artery MCFA medial circumflex femoral artery SFA superficial femoral artery

**Table 5:** Prevalence of the various types of CFA origins of the MCFA with respect to gender.

	Male			Female		
	Total	Right	Left	Total	Right	Left
Number of studies (number of legs)	5 (206)	4 (77)	4 (69)	5 (70)	4 (28)	4 (29)
From CFA (single trunk): % (95% CI)	80.4 (55.1–100)	66.2 (25.2–100)	73.4 (38.2–100)	84.4 (71.5–100)	76.1 (61.2–100)	73.4 (52.7–100)
From CFA (with DFA): % (95% CI)	13.5 (0–37.8)	21.5 (0–66.8)	17.8 (0–61.8)	6.7 (0–23.9)	9.3 (0–32.0)	10.8 (0–40.8)
From CFA (with LCFA): % (95% CI)	2.3 (0–15.7)	3.5 (0–31.5)	2.2 (0–27.1)	3.3 (0–16.7)	5.5 (0–24.3)	5.6 (0–29.9)
From CFA (with DFA and LCFA): % (95% CI)	1.8 (0–14.4)	4.4 (0–34.1)	2.2 (0–27.1)	1.9 (0–12.9)	3.0 (0–18.3)	3.4 (0–23.9)
From CFA (with DFA, LCFA and SFA): % (95% CI)	1.0 (0–11.6)	2.2 (0–27.1)	2.2 (0–27.1)	1.9 (0–12.9)	3.0 (0–18.3)	3.4 (0–23.9)
From CFA (with EPA): % (95% CI)	1.0 (0–11.6)	2.2 (0–27.1)	2.2 (0–27.1)	1.9 (0–12.9)	3.0 (0–18.3)	3.4 (0–23.9)
*I*^2^: % (95% CI)	92.69 (85.92–96.28)	88.54 (73.20–95.10)	87.85 (71.22–94.87)	77.35 (45.26–90.63)	57.12 (0–85.77)	65.24 (0–88.19)
Cochran’s q, *p*-value	<0.001	<0.001	<0.001	0.001	0.072	0.035

**Notes.**

CFA common femoral artery DFA deep femoral artery EPA external pudendal artery LCFA lateral circumflex femoral artery MCFA medial circumflex femoral artery SFA superficial femoral artery

Data on the prevalence of the various origins of the MCFA according to side were also extracted (Table 6). Our results showed that, like our overall analysis, the MCFA most commonly originated from the DFA. However, the prevalence of a DFA origin MCFA was slightly more common on the left side with a prevalence of 69.4% (95% CI [65.1–76.6]) versus the right side which had a prevalence of 62.4% (95% CI [55.2–72.2]). On the other hand, the MCFA originating from the CFA, which was the second most common origin, was more commonly found on the right side (34.6%) versus the left side (28.2%). However, due to overlapping confidence intervals, these differences were not statistically significant. Further results on the various origins of the MCFA according to side can be found in Tables 6 and 7.

**Table 6:** Prevalence of the various origins of the MCFA with respect to side.

	Right	Left
Number of studies (number of legs)	10 (704)	10 (705)
From CFA: % (95% CI)	34.6 (27.2–44.2)	28.2 (23.2–34.7)
From SFA: % (95% CI)	1.5 (0–4.7)	1.1 (0.1–2.9)
From DFA: % (95% CI)	62.4 (55.2–72.2)	69.4 (65.1–76.6)
From LCFA: % (95% CI)	0.5 (0–2.1)	0.4 (0–1.5)
From EIA: % (95% CI)	0.5 (0–2.1)	0.5 (0–1.7)
Aplasia: % (95% CI)	0.5 (0–2.1)	0.4 (0–1.5)
*I*^2^: % (95% CI)	78.76 (61.40–88.31)	57.83 (14.90–79.11)
Cochran’s q, *p*-value	<0.001	0.011

**Notes.**

CFA common femoral artery DFA deep femoral artery EIA external iliac artery LCFA lateral circumflex femoral artery MCFA medial circumflex femoral artery SFA superficial femoral artery

**Table 7:** Prevalence of the various types of CFA origins of the MCFA with respect to side.

	Right	Left
Number of studies (number of legs)	10 (232)	10 (198)
From CFA (single trunk): % (95% CI)	77.2 (60.4–95.1)	79.5 (64.9–97.6)
From CFA (with DFA): % (95% CI)	15.8 (3.1–35.8)	13.8 (2.0–34.1)
From CFA (with LCFA): % (95% CI)	2.4 (0–11.4)	1.9 (0–10.6)
From CFA (with DFA and LCFA): % (95% CI)	1.9 (0–10.2)	1.6 (0–9.9)
From CFA (with DFA, LCFA and SFA): % (95% CI)	1.4 (0–9.0)	1.6 (0–9.9)
From CFA (with EPA): % (95% CI)	1.4 (0–9.0)	1.6 (0–9.9)
*I*^2^: % (95% CI)	89.76 (83.30–93.72)	88.36 (80.67–92.99)
Cochran’s q, *p*-value	<0.001	<0.001

**Notes.**

CFA common femoral artery DFA deep femoral artery EPA external pudendal artery LCFA lateral circumflex femoral artery MCFA medial circumflex femoral artery SFA superficial femoral artery

### Pooled mean distance of the medial circumflex femoral artery origin to the mid-inguinal point

A total of 4 studies (*n* = 149 lower limbs) reported data on the pooled mean distance of the MCFA originating from the DFA to the mid-inguinal point (MIP) (Table 8). Our results showed that the pooled mean distance was 50.14 mm (95% CI [42.50–57.78]). This was longer than the distance reported in the same 4 studies (*n* = 54 lower limbs) for the MCFA originating from the CFA to MIP, which has a pooled mean of only 30.58 mm (95% CI [21.52–39.20]).

**Table 8:** Pooled mean distance of the MCFA originating from the DFA and from the CFA to the MIP.

	From the DFA	From the CFA
Number of studies (number of legs)	4 (149)	4 (54)
Pooled mean distance (mm): % (95% CI)	50.14 (42.50–57.78)	30.58 (21.52–39.20)
*I*^2^: %	33.09	0.0

**Notes.**

CFA common femoral artery DFA deep femoral artery MCFA medial circumflex femoral artery MIP mid-inguinal point

### New classification system for origin of the MCFA

**Figure 4:**
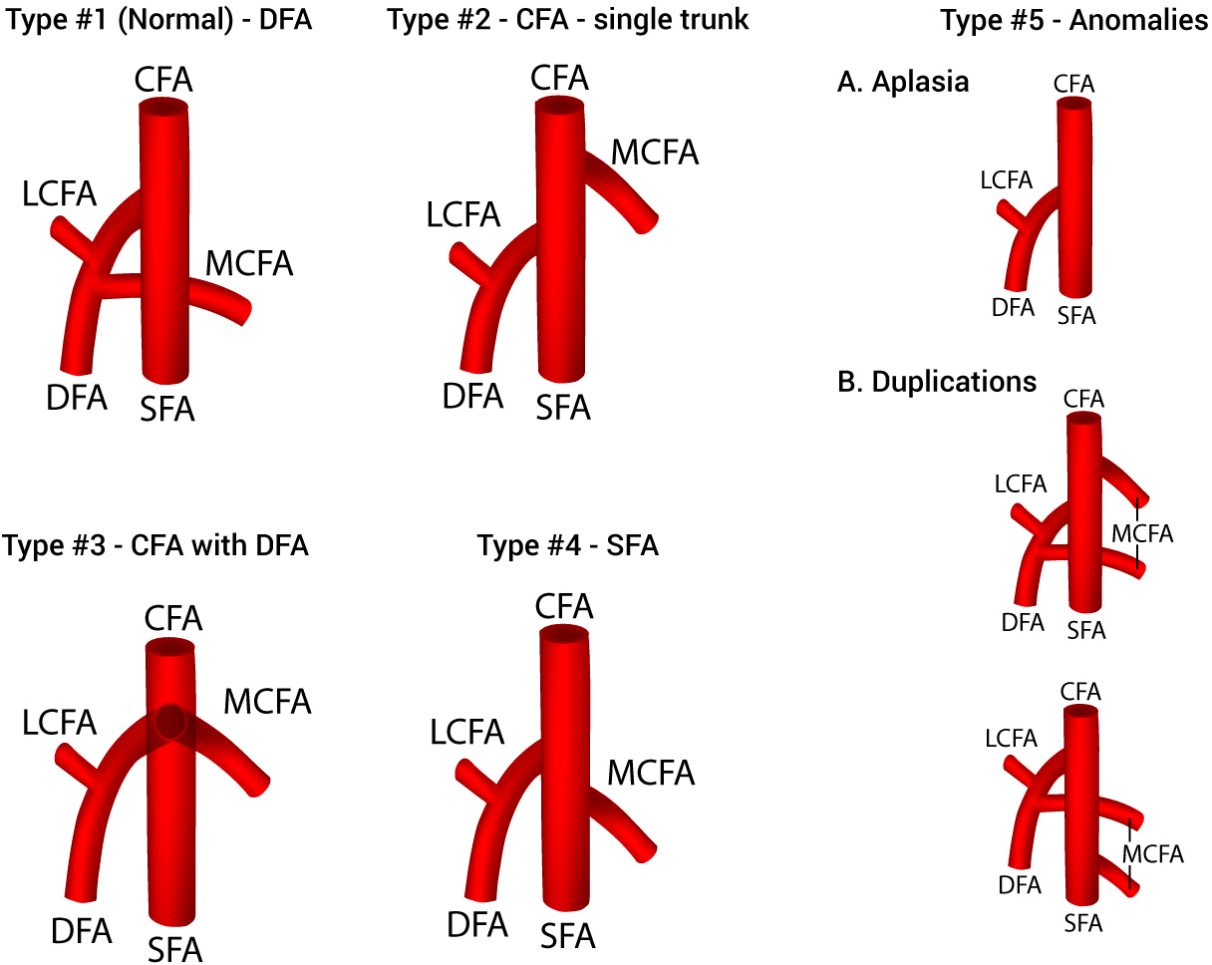
A new classification system of the origin of the medial circumflex femoral artery. CFA, common femoral artery; DFA, deep femoral artery; LCFA, lateral circumflex femoral artery; MCFA, medial circumflex femoral artery; SFA, superficial femoral artery.

After a thorough assessment of the results of the analysis, a new classification system for the origin of the MCFA was established and presented in Fig. 4. Five different types of variations that met the a priori thresholds were included in the classification: Type 1 (normal)—MCFA branching from the DFA; Type 2—MCFA branching from the CFA as a single trunk; Type 3—MCFA branching from the CFA with the DFA; Type 4—MCFA branching from the SFA; and Type 5—Anomalies (MCFA—A. Aplasia, B. Duplications). Although two distinct types of duplication patterns were identified, for the purposes of a clinically applicable classification system, all duplication patterns of MCFA origin are considered as on one type (5B).

## Discussion

Variations within the MCFA and its branches have been attributed to variability of blood flow through the rete femorale throughout embryogenesis (Perera, 1995; Sahin et al., 2003), however, the prevalence of origination patterns is not entirely understood. The overall aim of our study was to gather and analyze all available data from a comprehensive literature search on the MCFA to provide an evidence-based review of its anatomy that is essential for both clinical and surgical practice. Knowledge of the origin of the MCFA is a vital detail for surgeons with numerous implications. Accurate data regarding the artery may help reduce the incidence of avascular necrosis of the femoral head during embolization and hip surgery (Kalhor et al., 2009; Oide, 1979). For example, the KL approach, a common orthopedic technique used in acetabular fractures, poses a significant risk of vascular iatrogenic injury to the MCFA, especially when coupled with a poor understanding of the artery’s anatomy (Freitas et al., 2012).

Our results found that the MCFA most commonly originates from the DFA, regardless of geographical distribution, gender, or limb side, with an overall pooled prevalence of 64.6% (Tables 2, 4 and 6). Thus, we consider a DFA origin to be the normal type of MCFA origin.

In addition to a DFA origin, we identified 7 other variants, with pooled prevalences ranging from 0.4% to 32.2%. Such diverse findings emphasize the high variability of the MCFA origin. In order to provide some clinical clarity to the various origination patterns of the MCFA, we formulated a simple classification system (Fig. 4). The system is inclusive of all variants with a population prevalence ≥1.0%, and organized as most common—Type 1 (DFA origin) to least common—Type 4 (SFA origin). In addition, we formulated a Type 5, classified anomalies, for rare variants with a prevalence ≥0.5%.

The second most common origin of the MCFA was from the CFA (32.2%). This value was lower in the North American subgroup (28.9%) and found to be higher in the African and Asian subgroups (37.9% and 30.9%, respectively). Unlike the DFA type origin, there was a much larger amount of diversity to the branching pattern when the MCFA was derived from the CFA, thus several sub-variants were included for a separate analysis. When branching from the CFA, the MCFA was found most commonly to branch as a single trunk in 81.1% of individuals and as a branch alongside the DFA in 12.2% (Table 3). This information varies extensively throughout the literature, as the MCFA was reported to be derived as a single trunk sub-variant in 33.3% (Al-Talalwah 2015) to 100% (Emura et al. 1989) of individuals, whereas the MCFA was reported to branch alongside the DFA in anywhere from 0% (Massoud & Fletcher 1997) to 72.2% (Gościcka, Gielecki & Zietek 1990). Additionally, other sub-variants such as branching alongside the LCFA (1.7%), with the DFA and LCFA concurrently (2.5%), with the DFA, LCFA and SFA concurrently (1.4%), or with the external pudendal artery (1.1%) were found in our analysis (Table 3). Interesting to note is the branching patterns in Asians, where the MCFA originated as a single trunk in only 70.2% individuals, as compared to 81.1% of individuals in the overall analysis. The Asian subgroup pooled prevalence of MCFA originating from the CFA alongside the DFA was 25.5%, which is substantially higher than the overall pooled prevalence of 12.2% (Table 3).

The distance from the MCFA origin to the MIP was also analyzed, with a pooled mean distance of 50.14 mm when originating from the DFA, the most common origin of the artery (Table 8). However, when the MCFA originates from the CFA, which occurs in about 32.2% of the population, we found the distance from the origin to the MIP to be significantly shorter than that of the DFA origin, with a pooled mean of 30.58 mm. This finding is both clinically and statistically relevant. Physicians must be aware of an MCFA originating from the CFA, as this high origin of the artery has been associated with increased risk of MCFA puncture during cardiac interventional procedures and with iatrogenic injuries during femoral vein phlebotomy in infants (Shiny Vinila et al., 2013). Furthermore, a high originating MCFA may alter a surgeon’s perception of their location with respect to the major vasculature, leading them to believe they are farther away from important vessels than they actually are Craxford, Gale & Lammin, (2013).

However, while these morphometric findings were statistically significant, they were based on only 4 studies with small sample sizes. While it is most certain that a CFA origin occurs more proximal than a DFA origin, further studies are needed to determine more precise measurements of population values, as these morphometrics likely vary between geographical regions and may be of value to interventional radiologists and orthopedic surgeons.

Our study was limited by the lack of a quality assessment tool and risk of bias assessment method for anatomical studies, as well as a lack of a proper measure for publication bias in multi-categorical pooled prevalence meta-analysis. Furthermore, our meta-analysis was also limited by the high heterogeneity among the included studies. However, despite extensive subgroup analysis by study type, geography, gender, and side, the heterogeneity persisted throughout the meta-analysis. The predominance of Asian (15 studies, *n* =1,484) and European (10 studies, *n* =1,242) studies, and North American (8 studies, *n* = 964), compared to the relative lack of African (3 studies, *n* = 479), and South American (1 study, *n* = 92) studies, may have slightly skewed the results from the true population prevalence. More studies are needed from Oceania, as well as Africa and South America, to study the prevalence trends across populations.

The methodology of the included studies does not appear to have played any role in the found heterogeneity with respect to MCFA origin findings. While all studies except for two used cadaveric data (Massoud & Fletcher, 1997; Gościcka, Gielecki & Zietek, 1990), the imaging subgroup results were not significantly different from the overall analysis. As such, we attribute the high heterogeneity to the naturally high variability of the MCFA. Lastly, the new proposed classification system for MCFA origin should be further evaluated in future original anatomical studies to assess its validity and utility.

## Conclusion

In conclusion, the most common reported origin of the MCFA among the included studies, is a single trunk from the DFA (Type 1), branching at an average distance of 50.14 mm from the MIP. However, the origination of the MCFA is a highly variable trait among the general population, and thus with the data available from our analysis, a new classification for the origin of the MCFA was proposed. Accurate knowledge about these anatomical properties convey important information to surgeons, especially during orthopedic interventions in the region of the hip, in which improperly conducted procedures may lead to iatrogenic injury of the MCFA with subsequent avascular necrosis of the femoral head.

## Supplemental Information

10.7717/peerj.1726/supp-1 Data S1 MCFA raw data

10.7717/peerj.1726/supp-2 Supplemental Information 1 PRISMA 2009 checklist

10.7717/peerj.1726/supp-3 Supplemental Information 2 Forrest plots for the origins of the medial circumflex femoral artery

## Additional Information and Declarations

### Data Availability

The following information was supplied regarding data availability: The raw data was supplied as Data S1.
