# Supplementary material for: The origin of the medial circumflex femoral artery: a meta-analysis and proposal of a new classification system
Source: PeerJ. 2016 Feb 29;4:e1726. doi: 10.7717/peerj.1726 (PMC4782729; doi:10.7717/peerj.1726)
Supplement: Data S1 [file peerj-04-1726-s001.pdf]

				Both Sexes, Both Sides																
					Common femoral artery (CFA)										Deep femoral artery (DFA)					
Study ID	Continent	Country	Modality	BOTH SEXES, BOTH SIDES n = (number of legs)	CFA (total)	CFA (single trunk)	CFA (with DFA)	CFA (with LCFA)	CFA (with LCFA and DFA)	CFA (with SFA, DFA and LCFA)	CFA (with EPA)	Duplicated. One from CFA, one from DFA	Duplicated. One from SFA, one from DFA	SFA	DFA (total)	DFA (single trunk)	DFA (with LCFA)	LCFA	External iliac	Aplasia
Al-Talalwah 2015	Europe	Scotland	Cadaveric	342	135	45	50	7		31	2	0	0	8	195	172	23	2	0	2
Gautier 2000	Europe	Switzerland	Cadaveric	24	4							0	0	0	20			0	0	0
Kalhor 2012	Asia	Iran	Cadaveric	35	12							0	0	0	23			0	0	0
Manjappa 2014	Asia	India	Cadaveric	39	19	16	3	0		0	0	0	0	0	20	20	0	0	0	0
Massoud 1997	North America	USA	Digital subtraction transfemoral aortogram	188	13	11	0	2	0	0	0	0	0	12	157	157	0	0	0	6
Nasr 2014	Asia	Saudi Arabia	Cadaveric	90	29	14	15	0	0	0	0	0	0	8	53	53	0	0	0	0
Prakash 2010	Asia	India	Cadaveric	64	21							0	0	0	43			0	0	0
Tanyeli 2006	Europe	Turkey	Cadaveric	100	17	15	1	0	1	0	0	4	0	0	79	79	0	0	0	0
Vazquez 2007	Europe	England	Cadaveric	438	106	68	0	2	36	0	0	0	0	2	330	285	45	0	0	0
Dixit 2001	Asia	India	Cadaveric	48	18	10	8	0	0	0	0	0	0	0	30	30	0	0	0	0
Dixit 2011	Asia	India	Cadaveric	228	73	38	35	0	0	0	0	0	0	15	140	140	0	0	0	0
Peera 2013	Asia	India	Cadaveric	40	10	8	2	0	0	0	0	0	0	0	30	30	0	0	0	0
Clarke 1993	North America	USA	Cadaveric	30	13	12	1	0	0	0	0	0	0	1	15	15	0	0	1	0
Keen 1961	Africa	South Africa	Cadaveric	280	106	106	0	0	0	0	0	0	0	0	174			0	0	0
Shiny Vinila 2013	Asia	India	Cadaveric	40	13	6	7	0	0	0	0	0	0	0	27	27	0	0	0	0
Lalovic 2013	Europe	Serbia	Cadaveric	42	15	14	1	0	0	0	0	0	0	0	25	25	0	0	0	2
Goscicka 1990	Europe	Poland	Imaging (radiogram)	100	36	10	26	0	0	0	0	0	0	0	64	0	64	0	0	0
Marcade 1978	Europe	France	Cadaveric	50	23	23	0	0	0	0	0	0	0	0	27	27	0	0	0	0
Emura 1989	Asia	Japan	Cadaveric	337	271	271	0	0	0	0	0	0	0	0	66	66	0	0	0	0
Munteanu 1998	Europe	Romania	Cadaveric	50	10	10	0	0	0	0	0	0	0	5	35	35	0	0	0	0
O'Hara 1983	Africa	South Africa	Cadaveric	19	5	5	0	0	0	0	0	0	0	0	14	14	0	0	0	0
Zlotorowicz 2013	Europe	Poland	Cadaveric	16	3	3	0	0	0	0	0	0	0	0	13	13	0	0	0	0
Anwer 2013	Asia	India	Cadaveric	60	16	13	3	0	0	0	0	0	0	0	44	44	0	0	0	0
Boonkham 1987	Asia	Thailand	Cadaveric	113	26	20	5	0	1	0	0	0	0	0	87	87	0	0	0	0
Gremigni 1968	Europe	Italy	Cadaveric	100	27	27	0	0	0	0	0	0	0	0	73	73	0	0	0	0
Siddharth 1985	North America	USA	Cadaveric	100	5	5	0	0	0	0	0	0	1	31	63	63	0	0	0	0
DeBeer 1965	Africa	South Africa	Cadaveric	180	72	70	0	2	0	0	0	0	0	0	108	108	0	0	0	0
Williams 1934	North America	USA	Cadaveric	481	135	131	0	4	0	0	0	0	0	0	346	346	0	0	0	0
Ming-Tzu 1937	Asia	China	Cadaveric	150	30	30	0	0	0	0	0	0	0	0	120	120	0	0	0	0
del Sol 2011	South America	Chile	Cadaveric	92	50	43	7	0	0	0	0	0	0	0	41	41	0	1	0	0
Darji 2015	Asia	India	Cadaveric	130	14	3	11	0	0	0	0	0	0	0	116	116	0	0	0	0
Chung 1976	North America	USA	Cadaveric	20	14							0	0	0	6			0	0	0
Lipschutz 1918	North America	USA	Cadaveric	95	76	76	0	0	0	0	0	0	0	0	19	19	0	0	0	0
Odgen 1974	North America	USA	Cadaveric	36	3							0	0	0	33			0	0	0
Bloda 1982	Europe	Poland	Cadaveric	80	50	12	12	0	26	0	0	0	0	0	29	29	0	0	0	1
Boraiah 2009	North America	USA	Cadaveric	14	2							0	0	0	12			0	0	0
Liu 2001	Asia	China	cadaveric	50	13							0	0	0	37	0	0	0	0	0
Luo 1997	Asia	China	cadaveric	50	17							0	0	0	33	0	0	0	0	0

				BOTH SEXES, Right Side														
					Common femoral artery (CFA)								Deep femoral artery (DFA)					
Study ID	Continent	Country	Modality	BOTH SEXES RIGHT SIDE n = (number of legs)	CFA (total)	CFA (single trunk)	CFA (with DFA)	CFA (with LCFA)	CFA (with LCFA and DFA)	CFA (with SFA, DFA and LCFA)	CFA (with EPA)	SFA	DFA (total)	DFA (single trunk)	DFA (with LCFA)	LCFA	External iliac	Aplasia
Al-Talalwah 2015	Europe	Scotland	Cadaveric															
Gautier 2000	Europe	Switzerland	Cadaveric															
Kalhor 2012	Asia	Iran	Cadaveric															
Manjappa 2014	Asia	India	Cadaveric	19	11	9	2	0		0	0	0	8	8	0	0	0	0
Massoud 1997	North America	USA	Digital subtraction transfemoral aortogram															
Nasr 2014	Asia	Saudi Arabia	Cadaveric	45	16	8	8	0	0	0	0	4	25	25	0	0	0	0
Prakash 2010	Asia	India	Cadaveric															
Tanyeli 2006	Europe	Turkey	Cadaveric															
Vazquez 2007	Europe	England	Cadaveric															
Dixit 2001	Asia	India	Cadaveric	24	12	7	5	0	0	0	0	0	12	12	0	0	0	0
Dixit 2011	Asia	India	Cadaveric	114	40	21	19	0	0	0	0	10	64	64	0	0	0	0
Peera 2013	Asia	India	Cadaveric															
Clarke 1993	North America	USA	Cadaveric	15	6	5	1	0	0	0	0	1	8	8	0	0	0	0
Keen 1961	Africa	South Africa	Cadaveric															
Shiny Vinila 2013	Asia	India	Cadaveric															
Lalovic 2013	Europe	Serbia	Cadaveric															
Goscicka 1990	Europe	Poland	Imaging (radiogram)															
Marcade 1978	Europe	France	Cadaveric															
Emura 1989	Asia	Japan	Cadaveric															
Munteanu 1998	Europe	Romania	Cadaveric															
O'Hara 1983	Africa	South Africa	Cadaveric															
Zlotorowicz 2013	Europe	Poland	Cadaveric															
Anwer 2013	Asia	India	Cadaveric	30	8	6	2	0	0	0	0	0	22	22	0	0	0	0
Boonkham 1987	Asia	Thailand	Cadaveric	56	15	12	2	0	1	0	0	0	41	41	0	0	0	0
Gremigni 1968	Europe	Italy	Cadaveric															
Siddharth 1985	North America	USA	Cadaveric															
DeBeer 1965	Africa	South Africa	Cadaveric	90	43	41	0	2	0	0	0	0	47	47	0	0	0	0
Williams 1934	North America	USA	Cadaveric	240	68	65	0	3	0	0	0	0	172	172	0	0	0	0
Ming-Tzu 1937	Asia	China	Cadaveric	76	14	14	0	0	0	0	0	0	62	62	0	0	0	0
del Sol 2011	South America	Chile	Cadaveric															
Darji 2015	Asia	India	Cadaveric	65	5	2	3	0	0	0	0	0	60	60	0	0	0	0
Chung 1976	North America	USA	Cadaveric															
Lipschutz 1918	North America	USA	Cadaveric	47	40	40	0	0	0	0	0	0	7	7	0	0	0	0
Odgen 1974	North America	USA	Cadaveric															
Bloda 1982	Europe	Poland	Cadaveric															
Boraiah 2009	North America	USA	Cadaveric															
Liu 2001	Asia	China	cadaveric															
Luo 1997	Asia	China	cadaveric															

				BOTH SEXES, LEFT SIDE														
					Common femoral artery (CFA)								Deep femoral artery (DFA)					
Study ID	Continent	Country	Modality	BOTH SEXES, LEFT SIDE n = (number of legs)	CFA (total)	CFA (single trunk)	CFA (with DFA)	CFA (with LCFA)	CFA (with LCFA and DFA)	CFA (with SFA, DFA and LCFA)	CFA (with EPA)	SFA	DFA (total)	DFA (single trunk)	DFA (with LCFA)	LCFA	External iliac	Aplasia
**Al-Talalwah 2015**	Europe	Scotland	Cadaveric															
**Gautier 2000**	Europe	Switzerland	Cadaveric															
**Kalhor 2012**	Asia	Iran	Cadaveric															
**Manjappa 2014**	Asia	India	Cadaveric	20	8	7	1	0		0	0	0	12	12	0	0	0	0
**Massoud 1997**	North America	USA	Digital subtraction transfemoral aortogram															
**Nasr 2014**	Asia	Saudi Arabia	Cadaveric	45	13	6	7	0	0	0	0	4	28	28	0	0	0	0
**Prakash 2010**	Asia	India	Cadaveric															
**Tanyeli 2006**	Europe	Turkey	Cadaveric															
**Vazquez 2007**	Europe	England	Cadaveric															
**Dixit 2001**	Asia	India	Cadaveric	24	6	3	3	0	0	0	0	0	18	18	0	0	0	0
**Dixit 2011**	Asia	India	Cadaveric	114	33	17	16	0	0	0	0	5	76	76	0	0	0	0
**Peera 2013**	Asia	India	Cadaveric															
**Clarke 1993**	North America	USA	Cadaveric	15	7	7	0	0	0	0	0	0	7	7	0	0	1	0
**Keen 1961**	Africa	South Africa	Cadaveric															
**Shiny Vinila 2013**	Asia	India	Cadaveric															
**Lalovic 2013**	Europe	Serbia	Cadaveric															
**Goscicka 1990**	Europe	Poland	Imaging (radiogram)															
**Marcade 1978**	Europe	France	Cadaveric															
**Emura 1989**	Asia	Japan	Cadaveric															
**Munteanu 1998**	Europe	Romania	Cadaveric															
**O'Hara 1983**	Africa	South Africa	Cadaveric															
**Zlotorowicz 2013**	Europe	Poland	Cadaveric															
**Anwer 2013**	Asia	India	Cadaveric	30	8	7	1	0	0	0	0	0	22	22	0	0	0	0
**Boonkham 1987**	Asia	Thailand	Cadaveric	57	11	8	3	0	0	0	0	0	46	46	0	0	0	0
**Gremigni 1968**	Europe	Italy	Cadaveric															
**Siddharth 1985**	North America	USA	Cadaveric															
**DeBeer 1965**	Africa	South Africa	Cadaveric	90	29	29	0	0	0	0	0	0	61	61	0	0	0	0
**Williams 1934**	North America	USA	Cadaveric	241	67	66	0	1	0	0	0	0	174	174	0	0	0	0
**Ming-Tzu 1937**	Asia	China	Cadaveric	74	16	16	0	0	0	0	0	0	58	58	0	0	0	0
**del Sol 2011**	South America	Chile	Cadaveric															
**Darji 2015**	Asia	India	Cadaveric	65	9	1	8	0	0	0	0	0	56	56	0	0	0	0
**Chung 1976**	North America	USA	Cadaveric															
**Lipschutz 1918**	North America	USA	Cadaveric	48	36	36	0	0	0	0	0	0	12	12	0	0	0	0
**Odgen 1974**	North America	USA	Cadaveric															
**Bloda 1982**	Europe	Poland	Cadaveric															
**Boraiah 2009**	North America	USA	Cadaveric															
Liu 2001	Asia	China	cadaveric															
Luo 1997	Asia	China	cadaveric															

				MALE, Both Sides														
					Common femoral artery (CFA)								Deep femoral artery (DFA)					
Study ID	Continent	Country	Modality	MALE BOTH SIDES n = (number of legs)	CFA (total)	CFA (single trunk)	CFA (with DFA)	CFA (with LCFA)	CFA (with LCFA and DFA)	CFA (with SFA, DFA and LCFA)	CFA (with EPA)	SFA	DFA (total)	DFA (single trunk)	DFA (with LCFA)	LCFA	External iliac	Aplasia
Al-Talalwah 2015	Europe	Scotland	Cadaveric															
Gautier 2000	Europe	Switzerland	Cadaveric															
Kalhor 2012	Asia	Iran	Cadaveric															
Manjappa 2014	Asia	India	Cadaveric															
Massoud 1997	North America	USA	Digital subtraction transfemoral aortogram															
Nasr 2014	Asia	Saudi Arabia	Cadaveric	50	16	7	9	0	0	0	0	4	30	30	0	0	0	0
Prakash 2010	Asia	India	Cadaveric															
Tanyeli 2006	Europe	Turkey	Cadaveric															
Vazquez 2007	Europe	England	Cadaveric															
Dixit 2001	Asia	India	Cadaveric															
Dixit 2011	Asia	India	Cadaveric															
Peera 2013	Asia	India	Cadaveric															
Clarke 1993	North America	USA	Cadaveric	18	10	9	1	0	0	0	0	1	6	6	0	0	1	0
Keen 1961	Africa	South Africa	Cadaveric															
Shiny Vinila 2013	Asia	India	Cadaveric															
Lalovic 2013	Europe	Serbia	Cadaveric															
Goscicka 1990	Europe	Poland	Imaging (radiogram)															
Marcade 1978	Europe	France	Cadaveric															
Emura 1989	Asia	Japan	Cadaveric															
Munteanu 1998	Europe	Romania	Cadaveric															
O'Hara 1983	Africa	South Africa	Cadaveric															
Zlotorowicz 2013	Europe	Poland	Cadaveric															
Anwer 2013	Asia	India	Cadaveric															
Boonkham 1987	Asia	Thailand	Cadaveric	74	13	7	5	0	1	0	0	0	61	61	0	0	0	0
Gremigni 1968	Europe	Italy	Cadaveric															
Siddharth 1985	North America	USA	Cadaveric															
DeBeer 1965	Africa	South Africa	Cadaveric	146	60	58	0	2	0	0	0	0	86	86	0	0	0	0
Williams 1934	North America	USA	Cadaveric	395	107	105	0	2	0	0	0	0	288	288	0	0	0	0
Ming-Tzu 1937	Asia	China	Cadaveric															
del Sol 2011	South America	Chile	Cadaveric															
Darji 2015	Asia	India	Cadaveric															
Chung 1976	North America	USA	Cadaveric															
Lipschutz 1918	North America	USA	Cadaveric															
Odgen 1974	North America	USA	Cadaveric															
Bloda 1982	Europe	Poland	Cadaveric															
Boraiah 2009	North America	USA	Cadaveric															
Liu 2001	Asia	China	cadaveric															
Luo 1997	Asia	China	cadaveric															

				MALE, RIGHT SIDE														
					Common femoral artery (CFA)								Deep femoral artery (DFA)					
**Study ID**	**Continent**	**Country**	**Modality**	**MALE, RIGHT SIDE n = (number of legs)**	**CFA (total)**	**CFA (single trunk)**	**CFA (with DFA)**	**CFA (with LCFA)**	**CFA (with LCFA and DFA)**	**CFA (with SFA, DFA and LCFA)**	**CFA (with EPA)**	**SFA**	**DFA (total)**	**DFA (single trunk)**	**DFA (with LCFA)**	**LCFA**	**External iliac**	**Aplasia**
**Al-Talalwah 2015**	Europe	Scotland	Cadaveric															
**Gautier 2000**	Europe	Switzerland	Cadaveric															
**Kalhor 2012**	Asia	Iran	Cadaveric															
**Manjappa 2014**	Asia	India	Cadaveric															
**Massoud 1997**	North America	USA	Digital subtraction transfemoral aortogram															
**Nasr 2014**	Asia	Saudi Arabia	Cadaveric	25	9	4	5	0	0	0	0	2	14	14	0	0	0	0
**Prakash 2010**	Asia	India	Cadaveric															
**Tanyeli 2006**	Europe	Turkey	Cadaveric															
**Vazquez 2007**	Europe	England	Cadaveric															
**Dixit 2001**	Asia	India	Cadaveric															
**Dixit 2011**	Asia	India	Cadaveric															
**Peera 2013**	Asia	India	Cadaveric															
**Clarke 1993**	North America	USA	Cadaveric	9	4	3	1	0	0	0	0	1	4	4	0	0	0	0
**Keen 1961**	Africa	South Africa	Cadaveric															
**Shiny Vinila 2013**	Asia	India	Cadaveric															
**Lalovic 2013**	Europe	Serbia	Cadaveric															
**Goscicka 1990**	Europe	Poland	Imaging (radiogram)															
**Marcade 1978**	Europe	France	Cadaveric															
**Emura 1989**	Asia	Japan	Cadaveric															
**Munteanu 1998**	Europe	Romania	Cadaveric															
**O'Hara 1983**	Africa	South Africa	Cadaveric															
**Zlotorowicz 2013**	Europe	Poland	Cadaveric															
**Anwer 2013**	Asia	India	Cadaveric															
**Boonkham 1987**	Asia	Thailand	Cadaveric	37	7	4	2	0	1	0	0	0	30	30	0	0	0	0
**Gremigni 1968**	Europe	Italy	Cadaveric															
**Siddharth 1985**	North America	USA	Cadaveric															
**DeBeer 1965**	Africa	South Africa	Cadaveric															
**Williams 1934**	North America	USA	Cadaveric	197	57	55	0	2	0	0	0	0	140	140	0	0	0	0
**Ming-Tzu 1937**	Asia	China	Cadaveric															
**del Sol 2011**	South America	Chile	Cadaveric															
**Darji 2015**	Asia	India	Cadaveric															
**Chung 1976**	North America	USA	Cadaveric															
**Lipschutz 1918**	North America	USA	Cadaveric															
**Odgen 1974**	North America	USA	Cadaveric															
**Bloda 1982**	Europe	Poland	Cadaveric															
**Boraiah 2009**	North America	USA	Cadaveric															
Liu 2001	Asia	China	cadaveric															
Luo 1997	Asia	China	cadaveric															

				MALE, LEFT SIDE														
					Common femoral artery (CFA)								Deep femoral artery (DFA)					
Study ID	Continent	Country	Modality	MALE, LEFT SIDE n = (number of legs)	CFA (total)	CFA (single trunk)	CFA (with DFA)	CFA (with LCFA)	CFA (with LCFA and DFA)	CFA (with SFA, DFA and LCFA)	CFA (with EPA)	SFA	DFA (total)	DFA (single trunk)	DFA (with LCFA)	LCFA	External iliac	Aplasia
Al-Talalwah 2015	Europe	Scotland	Cadaveric															
Gautier 2000	Europe	Switzerland	Cadaveric															
Kalhor 2012	Asia	Iran	Cadaveric															
Manjappa 2014	Asia	India	Cadaveric															
Massoud 1997	North America	USA	Digital subtraction transfemoral aortogram															
Nasr 2014	Asia	Saudi Arabia	Cadaveric	25	7	3	4	0	0	0	0	2	16	16	0	0	0	0
Prakash 2010	Asia	India	Cadaveric															
Tanyeli 2006	Europe	Turkey	Cadaveric															
Vazquez 2007	Europe	England	Cadaveric															
Dixit 2001	Asia	India	Cadaveric															
Dixit 2011	Asia	India	Cadaveric															
Peera 2013	Asia	India	Cadaveric															
Clarke 1993	North America	USA	Cadaveric	9	6	6	0	0	0	0	0	0	2	2	0	0	1	0
Keen 1961	Africa	South Africa	Cadaveric															
Shiny Vinila 2013	Asia	India	Cadaveric															
Lalovic 2013	Europe	Serbia	Cadaveric															
Goscicka 1990	Europe	Poland	Imaging (radiogram)															
Marcade 1978	Europe	France	Cadaveric															
Emura 1989	Asia	Japan	Cadaveric															
Munteanu 1998	Europe	Romania	Cadaveric															
O'Hara 1983	Africa	South Africa	Cadaveric															
Zlotorowicz 2013	Europe	Poland	Cadaveric															
Anwer 2013	Asia	India	Cadaveric															
Boonkham 1987	Asia	Thailand	Cadaveric	37	6	3	3	0	0	0	0	0	31	31	0	0	0	0
Gremigni 1968	Europe	Italy	Cadaveric															
Siddharth 1985	North America	USA	Cadaveric															
DeBeer 1965	Africa	South Africa	Cadaveric															
Williams 1934	North America	USA	Cadaveric	198	50	50	0	0	0	0	0	0	148	148	0	0	0	0
Ming-Tzu 1937	Asia	China	Cadaveric															
del Sol 2011	South America	Chile	Cadaveric															
Darji 2015	Asia	India	Cadaveric															
Chung 1976	North America	USA	Cadaveric															
Lipschutz 1918	North America	USA	Cadaveric															
Odgen 1974	North America	USA	Cadaveric															
Bloda 1982	Europe	Poland	Cadaveric															
Boraiah 2009	North America	USA	Cadaveric															
Liu 2001	Asia	China	cadaveric															
Luo 1997	Asia	China	cadaveric															

				FEMALE, Both Sides													
					Common femoral artery (CFA)								Deep femoral artery (DFA)				
Study ID	Continent	Country	Modality	FEMALE BOTH SIDES n = (number of legs)	CFA (total)	CFA (single trunk)	CFA (with DFA)	CFA (with LCFA)	CFA (with LCFA and DFA)	CFA (with SFA, DFA and LCFA)	CFA (with EPA)	SFA	DFA (total)	DFA (single trunk)	DFA (with LCFA)	LCFA	Aplasia
**Al-Talalwah 2015**	Europe	Scotland	Cadaveric														
**Gautier 2000**	Europe	Switzerland	Cadaveric														
**Kalhor 2012**	Asia	Iran	Cadaveric														
**Manjappa 2014**	Asia	India	Cadaveric														
**Massoud 1997**	North America	USA	Digital subtraction transfemoral aortogram														
**Nasr 2014**	Asia	Saudi Arabia	Cadaveric	40	13	7	6	0	0	0	0	4	23	23	0	0	0
**Prakash 2010**	Asia	India	Cadaveric														
**Tanyeli 2006**	Europe	Turkey	Cadaveric														
**Vazquez 2007**	Europe	England	Cadaveric														
**Dixit 2001**	Asia	India	Cadaveric														
**Dixit 2011**	Asia	India	Cadaveric														
**Peera 2013**	Asia	India	Cadaveric														
**Clarke 1993**	North America	USA	Cadaveric	12	3	3	0	0	0	0	0	0	9	9	0	0	0
**Keen 1961**	Africa	South Africa	Cadaveric														
**Shiny Vinila 2013**	Asia	India	Cadaveric														
**Lalovic 2013**	Europe	Serbia	Cadaveric														
**Goscicka 1990**	Europe	Poland	Imaging (radiogram)														
**Marcade 1978**	Europe	France	Cadaveric														
**Emura 1989**	Asia	Japan	Cadaveric														
**Munteanu 1998**	Europe	Romania	Cadaveric														
**O'Hara 1983**	Africa	South Africa	Cadaveric														
**Zlotorowicz 2013**	Europe	Poland	Cadaveric														
**Anwer 2013**	Asia	India	Cadaveric														
**Boonkham 1987**	Asia	Thailand	Cadaveric	39	13	13	0	0	0	0	0	0	26	26	0	0	0
**Gremigni 1968**	Europe	Italy	Cadaveric														
**Siddharth 1985**	North America	USA	Cadaveric														
**DeBeer 1965**	Africa	South Africa	Cadaveric	33	28	11	0	17	0	0	0	0	5	5	0	0	0
**Williams 1934**	North America	USA	Cadaveric	86	28	26	0	2	0	0	0	0	58	58	0	0	0
**Ming-Tzu 1937**	Asia	China	Cadaveric														
**del Sol 2011**	South America	Chile	Cadaveric														
**Darji 2015**	Asia	India	Cadaveric														
**Chung 1976**	North America	USA	Cadaveric														
**Lipschutz 1918**	North America	USA	Cadaveric														
**Odgen 1974**	North America	USA	Cadaveric														
**Bloda 1982**	Europe	Poland	Cadaveric														
**Boraiah 2009**	North America	USA	Cadaveric														
Liu 2001	Asia	China	cadaveric														
Luo 1997	Asia	China	cadaveric														

				FEMALE, Right Side													
					Common femoral artery (CFA)								Deep femoral artery (DFA)				
**Study ID**	**Continent**	**Country**	**Modality**	**FEMALE, RIGHT SIDE n = (number of legs)**	**CFA (total)**	**CFA (single trunk)**	**CFA (with DFA)**	**CFA (with LCFA)**	**CFA (with LCFA and DFA)**	**CFA (with SFA, DFA and LCFA)**	**CFA (with EPA)**	**SFA**	**DFA (total)**	**DFA (single trunk)**	**DFA (with LCFA)**	**LCFA**	**Aplasia**
**Al-Talalwah 2015**	Europe	Scotland	Cadaveric														
**Gautier 2000**	Europe	Switzerland	Cadaveric														
**Kalhor 2012**	Asia	Iran	Cadaveric														
**Manjappa 2014**	Asia	India	Cadaveric														
**Massoud 1997**	North America	USA	Digital subtraction transfemoral aortogram														
**Nasr 2014**	Asia	Saudi Arabia	Cadaveric	20	7	4	3	0	0	0	0	2	11	11	0	0	0
**Prakash 2010**	Asia	India	Cadaveric														
**Tanyeli 2006**	Europe	Turkey	Cadaveric														
**Vazquez 2007**	Europe	England	Cadaveric														
**Dixit 2001**	Asia	India	Cadaveric														
**Dixit 2011**	Asia	India	Cadaveric														
**Peera 2013**	Asia	India	Cadaveric														
**Clarke 1993**	North America	USA	Cadaveric	6	2	2	0	0	0	0	0	0	4	4	0	0	0
**Keen 1961**	Africa	South Africa	Cadaveric														
**Shiny Vinila 2013**	Asia	India	Cadaveric														
**Lalovic 2013**	Europe	Serbia	Cadaveric														
**Goscicka 1990**	Europe	Poland	Imaging (radiogram)														
**Marcade 1978**	Europe	France	Cadaveric														
**Emura 1989**	Asia	Japan	Cadaveric														
**Munteanu 1998**	Europe	Romania	Cadaveric														
**O'Hara 1983**	Africa	South Africa	Cadaveric														
**Zlotorowicz 2013**	Europe	Poland	Cadaveric														
**Anwer 2013**	Asia	India	Cadaveric														
**Boonkham 1987**	Asia	Thailand	Cadaveric	19	8	8	0	0	0	0	0	0	11	11	0	0	0
**Gremigni 1968**	Europe	Italy	Cadaveric														
**Siddharth 1985**	North America	USA	Cadaveric														
**DeBeer 1965**	Africa	South Africa	Cadaveric														
**Williams 1934**	North America	USA	Cadaveric	43	11	10	0	1	0	0	0	0	32	32	0	0	0
**Ming-Tzu 1937**	Asia	China	Cadaveric														
**del Sol 2011**	South America	Chile	Cadaveric														
**Darji 2015**	Asia	India	Cadaveric														
**Chung 1976**	North America	USA	Cadaveric														
**Lipschutz 1918**	North America	USA	Cadaveric														
**Odgen 1974**	North America	USA	Cadaveric														
**Bloda 1982**	Europe	Poland	Cadaveric														
**Boraiah 2009**	North America	USA	Cadaveric														
Liu 2001	Asia	China	cadaveric														
Luo 1997	Asia	China	cadaveric														

				FEMALE, LEFT SIDE													
					Common femoral artery (CFA)								Deep femoral artery (DFA)				
Study ID	Continent	Country	Modality	FEMALE, LEFT SIDE n = (number of legs)	CFA (total)	CFA (single trunk)	CFA (with DFA)	CFA (with LCFA)	CFA (with LCFA and DFA)	CFA (with SFA, DFA and LCFA)	CFA (with EPA)	SFA	DFA (total)	DFA (single trunk)	DFA (with LCFA)	LCFA	Aplasia
Al-Talalwah 2015	Europe	Scotland	Cadaveric														
Gautier 2000	Europe	Switzerland	Cadaveric														
Kalhor 2012	Asia	Iran	Cadaveric														
Manjappa 2014	Asia	India	Cadaveric														
Massoud 1997	North America	USA	Digital subtraction transfemoral aortogram														
Nasr 2014	Asia	Saudi Arabia	Cadaveric	20	6	3	3	0	0	0	0	2	12	12	0	0	0
Prakash 2010	Asia	India	Cadaveric														
Tanyeli 2006	Europe	Turkey	Cadaveric														
Vazquez 2007	Europe	England	Cadaveric														
Dixit 2001	Asia	India	Cadaveric														
Dixit 2011	Asia	India	Cadaveric														
Peera 2013	Asia	India	Cadaveric														
Clarke 1993	North America	USA	Cadaveric	6	1	1	0	0	0	0	0	0	5	5	0	0	0
Keen 1961	Africa	South Africa	Cadaveric														
Shiny Vinila 2013	Asia	India	Cadaveric														
Lalovic 2013	Europe	Serbia	Cadaveric														
Goscicka 1990	Europe	Poland	Imaging (radiogram)														
Marcade 1978	Europe	France	Cadaveric														
Emura 1989	Asia	Japan	Cadaveric														
Munteanu 1998	Europe	Romania	Cadaveric														
O'Hara 1983	Africa	South Africa	Cadaveric														
Zlotorowicz 2013	Europe	Poland	Cadaveric														
Anwer 2013	Asia	India	Cadaveric														
Boonkham 1987	Asia	Thailand	Cadaveric	20	5	5	0	0	0	0	0	0	15	15	0	0	0
Gremigni 1968	Europe	Italy	Cadaveric														
Siddharth 1985	North America	USA	Cadaveric														
DeBeer 1965	Africa	South Africa	Cadaveric														
Williams 1934	North America	USA	Cadaveric	43	17	16	0	1	0	0	0	0	26	26	0	0	0
Ming-Tzu 1937	Asia	China	Cadaveric														
del Sol 2011	South America	Chile	Cadaveric														
Darji 2015	Asia	India	Cadaveric														
Chung 1976	North America	USA	Cadaveric														
Lipschutz 1918	North America	USA	Cadaveric														
Odgen 1974	North America	USA	Cadaveric														
Bloda 1982	Europe	Poland	Cadaveric														
Boraiah 2009	North America	USA	Cadaveric														
Liu 2001	Asia	China	cadaveric														
Luo 1997	Asia	China	cadaveric														

							BOTH SEXES								
							BOTH SEXES, BOTH SIDES			BOTH SEXES, RIGHT SIDE			BOTH SEXES, LEFT SIDE		
Study ID	Continent	Country	Modality	Range (years)	From	To	n = (total number of legs)	Mean distance (mm)	SD (mm)	n = (right-sided legs)	Mean distance (mm)	SD (mm)	n = (left-sided legs)	Mean distance (mm)	SD (mm)
**Gautier 2000**	Europe	Switzerland	Cadaveric	60-80	From origin of deep branch of MCFA	Trochanteric crest (at level of lesser trochanter)	10	18.2	3.75	5	16.6	3	5	19.8	2
**Gautier 2000**	Europe	Switzerland	Cadaveric	60-80	From origin of deep branch of MCFA	Trochanteric crest (at level of insertion of obturator externus)	10	8.8	3	5	6.8	2.25	5	10.6	2.75
**Gautier 2000**	Europe	Switzerland	Cadaveric	60-80	From origin of deep branch of MCFA	Trochanteric crest (at level of insertion of obturator internus)	10	12.4	2.5	5	12	2.5	5	12.8	1.25
**Freitas 2012**	South America	Brazil	Cadaveric		Origin of MCFA	Superolateral insertion of quadratus femoris	12	19.58	1.5	6	19	1.25	6	20.16	1
**Tanyeli 2006**	Europe	Turkey	Cadaveric	24-78	From MCFA (originating from DFA)	Mid-inguinal point (MIP)	79	59	15						
**Tanyeli 2006**	Europe	Turkey	Cadaveric	24-78	From MCFA (originating from CFA)	Mid-inguinal point (MIP)	17	25	14						
**Peera 2013**	Asia	India	Cadaveric		From MCFA (originating from DFA)	Mid-inguinal point (MIP)	30	36.8	17.5						
**Peera 2013**	Asia	India	Cadaveric		From MCFA (originating from CFA)	Mid-inguinal point (MIP)	10	26.8	17.5						
**Clarke 1993**	North America	USA	Cadaveric	49-85	From MCFA (originating from DFA)	Mid-inguinal point (MIP)	14	48.3	9.5	7	49.7	9.5	7	46.9	5.5
**Clarke 1993**	North America	USA	Cadaveric	49-85	From MCFA (originating from CFA)	Mid-inguinal point (MIP)	13	40.8	7.5	6	43	7.5	7	39	6.8
**Shiny Vinila 2013**	Asia	India	Cadaveric		From MCFA (originating from DFA)	MId-inguinal point (MIP)	26	54.8	7.5	14	54.8	6.75	12	54.3	6.75
**Shiny Vinila 2013**	Asia	India	Cadaveric		From MCFA (originating from CFA)	MId-inguinal point (MIP)	14	27.3	10	7	31.7	10	7	22.3	5

							MALE								
							MALE, BOTH SIDES			MALE, RIGHT SIDE			MALE, LEFT SIDE		
Study ID	Continent	Country	Modality	Range (years)	From	To	n = (total number of legs)	Mean distance (mm)	SD (mm)	n = (right-sided legs)	Mean distance (mm)	SD (mm)	n = (left-sided legs)	Mean distance (mm)	SD (mm)
**Gautier 2000**	Europe	Switzerland	Cadaveric	60-80	From origin of deep branch of MCFA	Trochanteric crest (at level of lesser trochanter)									
**Gautier 2000**	Europe	Switzerland	Cadaveric	60-80	From origin of deep branch of MCFA	Trochanteric crest (at level of insertion of obturator externus)									
**Gautier 2000**	Europe	Switzerland	Cadaveric	60-80	From origin of deep branch of MCFA	Trochanteric crest (at level of insertion of obturator internus)									
**Freitas 2012**	South America	Brazil	Cadaveric		Origin of MCFA	Superolateral insertion of quadratus femoris	6	20.83	0.5	3	20.33	0.25	3	21.33	0.25
**Tanyeli 2006**	Europe	Turkey	Cadaveric	24-78	From MCFA (originating from DFA)	Mid-inguinal point (MIP)									
**Tanyeli 2006**	Europe	Turkey	Cadaveric	24-78	From MCFA (originating from CFA)	Mid-inguinal point (MIP)									
**Peera 2013**	Asia	India	Cadaveric		From MCFA (originating from DFA)	Mid-inguinal point (MIP)									
**Peera 2013**	Asia	India	Cadaveric		From MCFA (originating from CFA)	Mid-inguinal point (MIP)									
**Clarke 1993**	North America	USA	Cadaveric	49-85	From MCFA (originating from DFA)	Mid-inguinal point (MIP)	5	50.8	5	3	50.7	5	2	51	3
**Clarke 1993**	North America	USA	Cadaveric	49-85	From MCFA (originating from CFA)	Mid-inguinal point (MIP)	10	38.6	6.8	4	37	6	6	39.7	6.8
**Shiny Vinila 2013**	Asia	India	Cadaveric		From MCFA (originating from DFA)	MId-inguinal point (MIP)									
**Shiny Vinila 2013**	Asia	India	Cadaveric		From MCFA (originating from CFA)	MId-inguinal point (MIP)									

							FEMALE								
							FEMALE, BOTH SIDES			FEMALE, RIGHT SIDE			FEMALE, LEFT SIDE		
Study ID	Continent	Country	Modality	Range (years)	From	To	n = (total number of legs)	Mean distance (mm)	SD (mm)	n = (right-sided legs)	Mean distance (mm)	SD (mm)	n = (left-sided legs)	Mean distance (mm)	SD (mm)
**Gautier 2000**	Europe	Switzerland	Cadaveric	60-80	From origin of deep branch of MCFA	Trochanteric crest (at level of lesser trochanter)									
**Gautier 2000**	Europe	Switzerland	Cadaveric	60-80	From origin of deep branch of MCFA	Trochanteric crest (at level of insertion of obturator externus)									
**Gautier 2000**	Europe	Switzerland	Cadaveric	60-80	From origin of deep branch of MCFA	Trochanteric crest (at level of insertion of obturator internus)									
**Freitas 2012**	South America	Brazil	Cadaveric		Origin of MCFA	Superolateral insertion of quadratus femoris	6	18.33	1	3	17.67	0.75	3	19	0.5
**Tanyeli 2006**	Europe	Turkey	Cadaveric	24-78	From MCFA (originating from DFA)	Mid-inguinal point (MIP)									
**Tanyeli 2006**	Europe	Turkey	Cadaveric	24-78	From MCFA (originating from CFA)	Mid-inguinal point (MIP)									
**Peera 2013**	Asia	India	Cadaveric		From MCFA (originating from DFA)	Mid-inguinal point (MIP)									
**Peera 2013**	Asia	India	Cadaveric		From MCFA (originating from CFA)	Mid-inguinal point (MIP)									
**Clarke 1993**	North America	USA	Cadaveric	49-85	From MCFA (originating from DFA)	Mid-inguinal point (MIP)	9	46.9	9.5	4	49	9.5	5	45.2	4.25
**Clarke 1993**	North America	USA	Cadaveric	49-85	From MCFA (originating from CFA)	Mid-inguinal point (MIP)	3	48.3	5						
**Shiny Vinila 2013**	Asia	India	Cadaveric		From MCFA (originating from DFA)	MId-inguinal point (MIP)									
**Shiny Vinila 2013**	Asia	India	Cadaveric		From MCFA (originating from CFA)	MId-inguinal point (MIP)									
