# Supplementary material for: The origin of the medial circumflex femoral artery: a meta-analysis and proposal of a new classification system
Source: PeerJ. 2016 Feb 29;4:e1726. doi: 10.7717/peerj.1726 (PMC4782729; doi:10.7717/peerj.1726)

From CFA

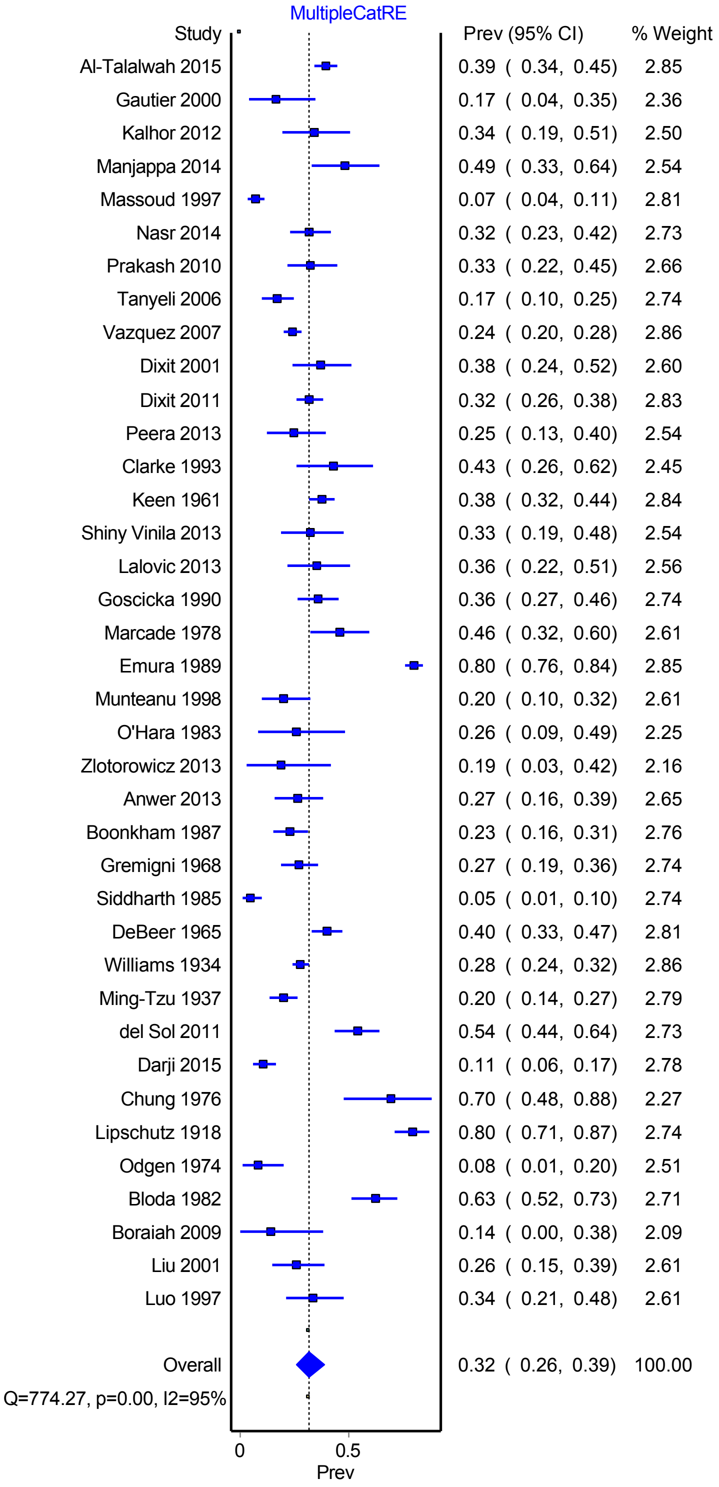
MultipleCatRE
Study
Prev (95% CI)
% Weight
Al-Talalwah 2015 0.39 (0.34, 0.45) 2.85
Gautier 2000 0.17 (0.04, 0.35) 2.36
Kalhor 2012 0.34 (0.19, 0.51) 2.50
Manjappa 2014 0.49 (0.33, 0.64) 2.54
Massoud 1997 0.07 (0.04, 0.11) 2.81
Nasr 2014 0.32 (0.23, 0.42) 2.73
Prakash 2010 0.33 (0.22, 0.45) 2.66
Tanyeli 2006 0.17 (0.10, 0.25) 2.74
Vazquez 2007 0.24 (0.20, 0.28) 2.86
Dixit 2001 0.38 (0.24, 0.52) 2.60
Dixit 2011 0.32 (0.26, 0.38) 2.83
Peera 2013 0.25 (0.13, 0.40) 2.54
Clarke 1993 0.43 (0.26, 0.62) 2.45
Keen 1961 0.38 (0.32, 0.44) 2.84
Shiny Vinila 2013 0.33 (0.19, 0.48) 2.54
Lalovic 2013 0.36 (0.22, 0.51) 2.56
Goscicka 1990 0.36 (0.27, 0.46) 2.74
Marcade 1978 0.46 (0.32, 0.60) 2.61
Emura 1989 0.80 (0.76, 0.84) 2.85
Munteanu 1998 0.20 (0.10, 0.32) 2.61
O'Hara 1983 0.26 (0.09, 0.49) 2.25
Zlotorowicz 2013 0.19 (0.03, 0.42) 2.16
Anwer 2013 0.27 (0.16, 0.39) 2.65
Boonkham 1987 0.23 (0.16, 0.31) 2.76
Gremigni 1968 0.27 (0.19, 0.36) 2.74
Siddharth 1985 0.05 (0.01, 0.10) 2.74
DeBeer 1965 0.40 (0.33, 0.47) 2.81
Williams 1934 0.28 (0.24, 0.32) 2.86
Ming-Tzu 1937 0.20 (0.14, 0.27) 2.79
del Sol 2011 0.54 (0.44, 0.64) 2.73
Darji 2015 0.11 (0.06, 0.17) 2.78
Chung 1976 0.70 (0.48, 0.88) 2.27
Lipschutz 1918 0.80 (0.71, 0.87) 2.74
Odgen 1974 0.08 (0.01, 0.20) 2.51
Bloda 1982 0.63 (0.52, 0.73) 2.71
Boraiah 2009 0.14 (0.00, 0.38) 2.09
Liu 2001 0.26 (0.15, 0.39) 2.61
Luo 1997 0.34 (0.21, 0.48) 2.61
Overall 0.32 (0.26, 0.39) 100.00
Q=774.27, p=0.00, I2=95%
0
0.5
Prev

Duplicated. One from DFA, one from CFA

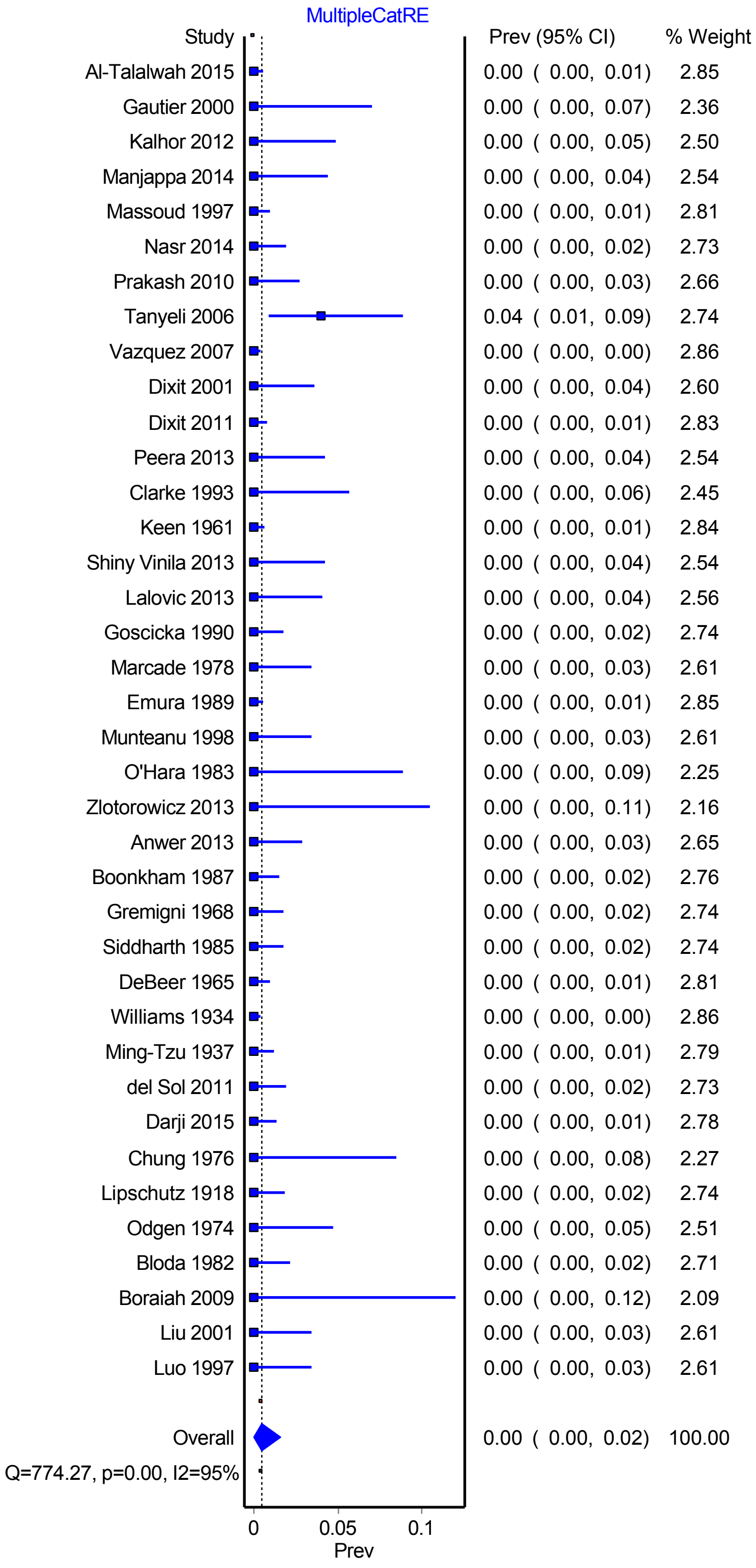

Duplicated. One from SFA, one from DFA

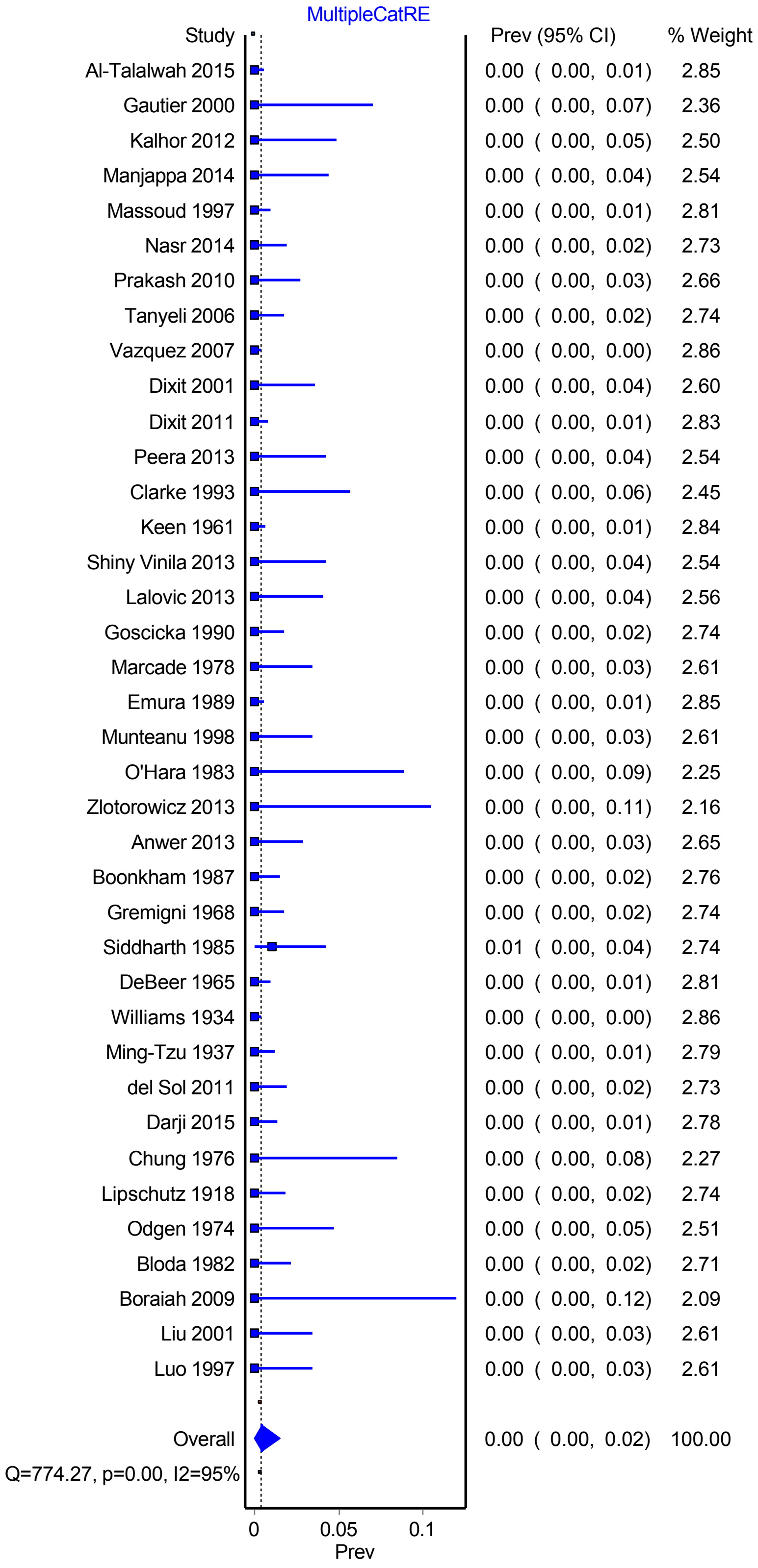

From SFA

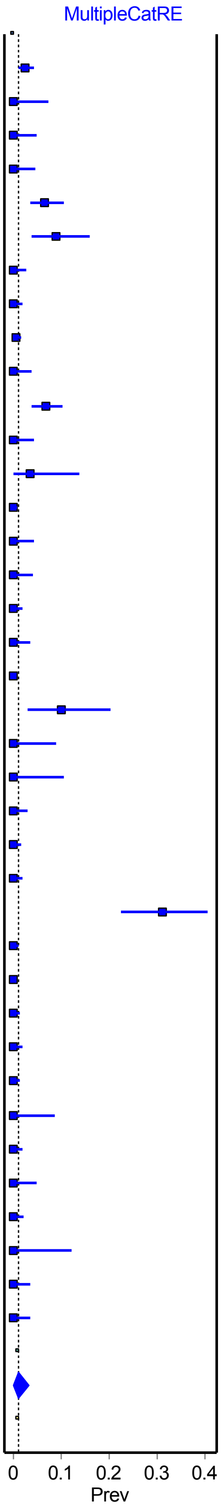

Study	Prev (95% CI)	% Weight
Al-Talalwah 2015	0.02 (0.01, 0.04)	2.85
Gautier 2000	0.00 (0.00, 0.07)	2.36
Kalhor 2012	0.00 (0.00, 0.05)	2.50
Manjappa 2014	0.00 (0.00, 0.04)	2.54
Massoud 1997	0.06 (0.03, 0.10)	2.81
Nasr 2014	0.09 (0.04, 0.16)	2.73
Prakash 2010	0.00 (0.00, 0.03)	2.66
Tanyeli 2006	0.00 (0.00, 0.02)	2.74
Vazquez 2007	0.00 (0.00, 0.01)	2.86
Dixit 2001	0.00 (0.00, 0.04)	2.60
Dixit 2011	0.07 (0.04, 0.10)	2.83
Peera 2013	0.00 (0.00, 0.04)	2.54
Clarke 1993	0.03 (0.00, 0.14)	2.45
Keen 1961	0.00 (0.00, 0.01)	2.84
Shiny Vinila 2013	0.00 (0.00, 0.04)	2.54
Lalovic 2013	0.00 (0.00, 0.04)	2.56
Goscicka 1990	0.00 (0.00, 0.02)	2.74
Marcade 1978	0.00 (0.00, 0.03)	2.61
Emura 1989	0.00 (0.00, 0.01)	2.85
Munteanu 1998	0.10 (0.03, 0.20)	2.61
O'Hara 1983	0.00 (0.00, 0.09)	2.25
Zlotorowicz 2013	0.00 (0.00, 0.11)	2.16
Anwer 2013	0.00 (0.00, 0.03)	2.65
Boonkham 1987	0.00 (0.00, 0.02)	2.76
Gremigni 1968	0.00 (0.00, 0.02)	2.74
Siddharth 1985	0.31 (0.22, 0.40)	2.74
DeBeer 1965	0.00 (0.00, 0.01)	2.81
Williams 1934	0.00 (0.00, 0.00)	2.86
Ming-Tzu 1937	0.00 (0.00, 0.01)	2.79
del Sol 2011	0.00 (0.00, 0.02)	2.73
Darji 2015	0.00 (0.00, 0.01)	2.78
Chung 1976	0.00 (0.00, 0.08)	2.27
Lipschutz 1918	0.00 (0.00, 0.02)	2.74
Odgen 1974	0.00 (0.00, 0.05)	2.51
Bloda 1982	0.00 (0.00, 0.02)	2.71
Boraiah 2009	0.00 (0.00, 0.12)	2.09
Liu 2001	0.00 (0.00, 0.03)	2.61
Luo 1997	0.00 (0.00, 0.03)	2.61
Overall	0.01 (0.00, 0.03)	100.00

Q=774.27, p=0.00, I2=95%

From DFA

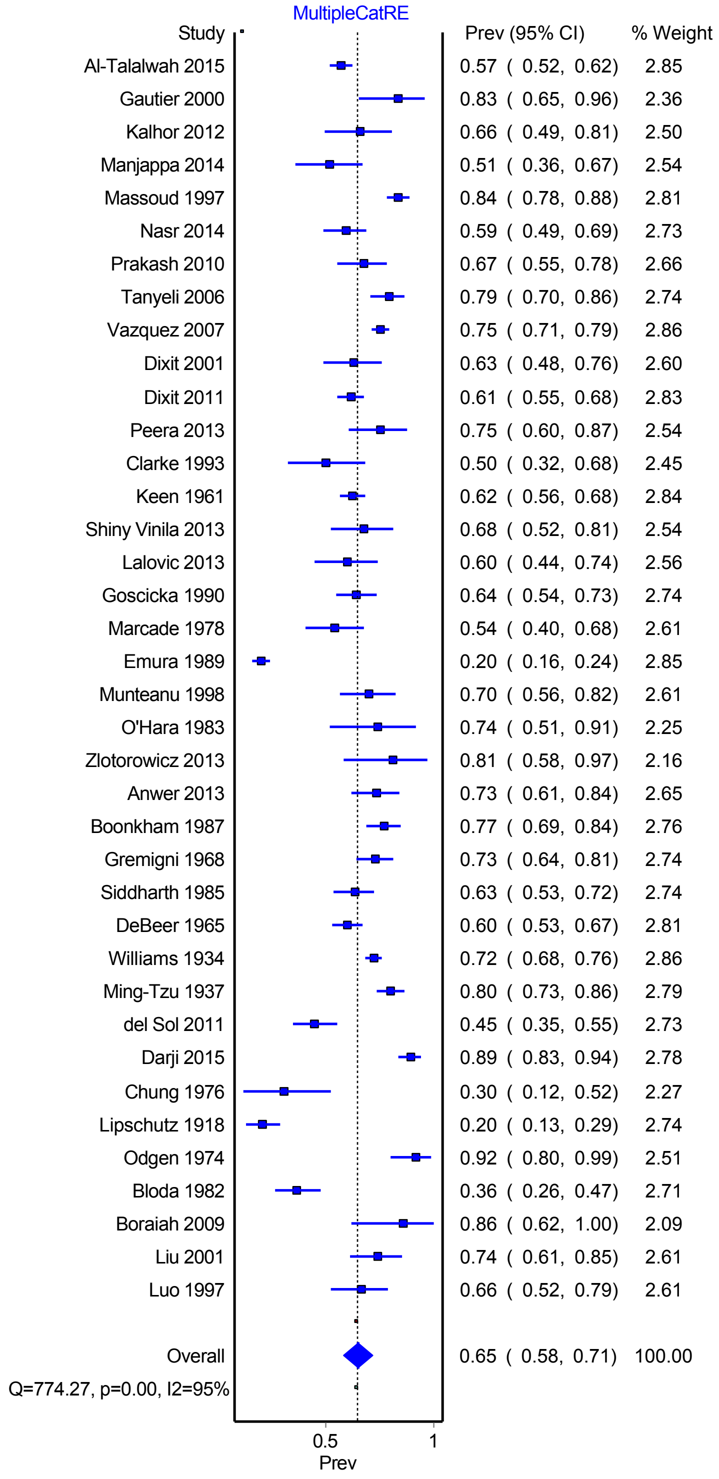
MultipleCatRE
Study
Prev (95% CI)
% Weight
Al-Talalwah 2015 0.57 (0.52, 0.62) 2.85
Gautier 2000 0.83 (0.65, 0.96) 2.36
Kalhor 2012 0.66 (0.49, 0.81) 2.50
Manjappa 2014 0.51 (0.36, 0.67) 2.54
Massoud 1997 0.84 (0.78, 0.88) 2.81
Nasr 2014 0.59 (0.49, 0.69) 2.73
Prakash 2010 0.67 (0.55, 0.78) 2.66
Tanyeli 2006 0.79 (0.70, 0.86) 2.74
Vazquez 2007 0.75 (0.71, 0.79) 2.86
Dixit 2001 0.63 (0.48, 0.76) 2.60
Dixit 2011 0.61 (0.55, 0.68) 2.83
Peera 2013 0.75 (0.60, 0.87) 2.54
Clarke 1993 0.50 (0.32, 0.68) 2.45
Keen 1961 0.62 (0.56, 0.68) 2.84
Shiny Vinila 2013 0.68 (0.52, 0.81) 2.54
Lalovic 2013 0.60 (0.44, 0.74) 2.56
Goscicka 1990 0.64 (0.54, 0.73) 2.74
Marcade 1978 0.54 (0.40, 0.68) 2.61
Emura 1989 0.20 (0.16, 0.24) 2.85
Munteanu 1998 0.70 (0.56, 0.82) 2.61
O'Hara 1983 0.74 (0.51, 0.91) 2.25
Zlotorowicz 2013 0.81 (0.58, 0.97) 2.16
Anwer 2013 0.73 (0.61, 0.84) 2.65
Boonkham 1987 0.77 (0.69, 0.84) 2.76
Gremigni 1968 0.73 (0.64, 0.81) 2.74
Siddharth 1985 0.63 (0.53, 0.72) 2.74
DeBeer 1965 0.60 (0.53, 0.67) 2.81
Williams 1934 0.72 (0.68, 0.76) 2.86
Ming-Tzu 1937 0.80 (0.73, 0.86) 2.79
del Sol 2011 0.45 (0.35, 0.55) 2.73
Darji 2015 0.89 (0.83, 0.94) 2.78
Chung 1976 0.30 (0.12, 0.52) 2.27
Lipschutz 1918 0.20 (0.13, 0.29) 2.74
Odgen 1974 0.92 (0.80, 0.99) 2.51
Bloda 1982 0.36 (0.26, 0.47) 2.71
Boraiah 2009 0.86 (0.62, 1.00) 2.09
Liu 2001 0.74 (0.61, 0.85) 2.61
Luo 1997 0.66 (0.52, 0.79) 2.61
Overall 0.65 (0.58, 0.71) 100.00
Q=774.27, p=0.00, I2=95%
0.5
1
Prev

From LCFA

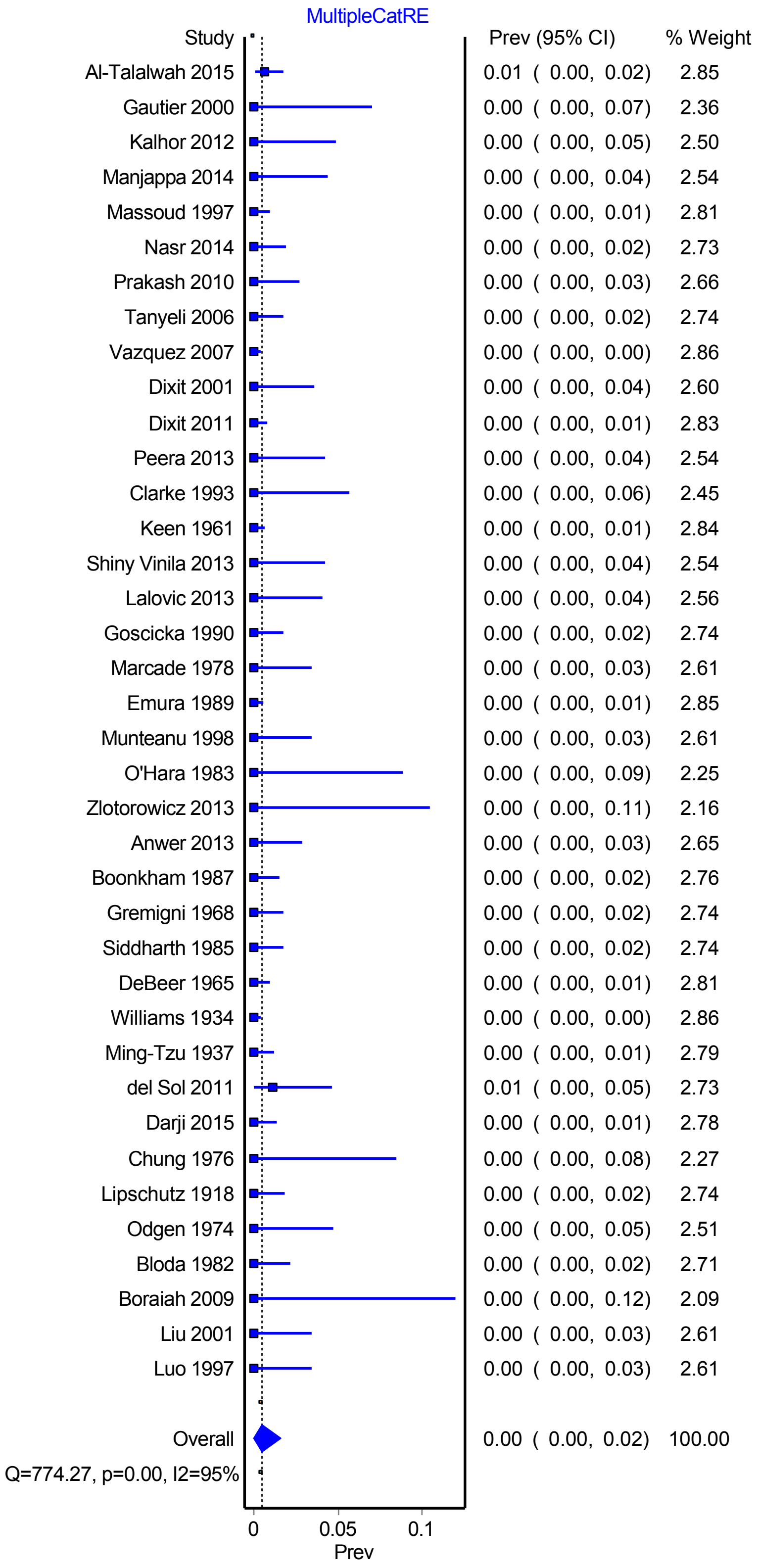
MultipleCatRE
Study
Prev (95% CI)
% Weight
Al-Talalwah 2015 0.01 (0.00, 0.02) 2.85
Gautier 2000 0.00 (0.00, 0.07) 2.36
Kalhor 2012 0.00 (0.00, 0.05) 2.50
Manjappa 2014 0.00 (0.00, 0.04) 2.54
Massoud 1997 0.00 (0.00, 0.01) 2.81
Nasr 2014 0.00 (0.00, 0.02) 2.73
Prakash 2010 0.00 (0.00, 0.03) 2.66
Tanyeli 2006 0.00 (0.00, 0.02) 2.74
Vazquez 2007 0.00 (0.00, 0.00) 2.86
Dixit 2001 0.00 (0.00, 0.04) 2.60
Dixit 2011 0.00 (0.00, 0.01) 2.83
Peera 2013 0.00 (0.00, 0.04) 2.54
Clarke 1993 0.00 (0.00, 0.06) 2.45
Keen 1961 0.00 (0.00, 0.01) 2.84
Shiny Vinila 2013 0.00 (0.00, 0.04) 2.54
Lalovic 2013 0.00 (0.00, 0.04) 2.56
Goscicka 1990 0.00 (0.00, 0.02) 2.74
Marcade 1978 0.00 (0.00, 0.03) 2.61
Emura 1989 0.00 (0.00, 0.01) 2.85
Munteanu 1998 0.00 (0.00, 0.03) 2.61
O'Hara 1983 0.00 (0.00, 0.09) 2.25
Zlotorowicz 2013 0.00 (0.00, 0.11) 2.16
Anwer 2013 0.00 (0.00, 0.03) 2.65
Boonkham 1987 0.00 (0.00, 0.02) 2.76
Gremigni 1968 0.00 (0.00, 0.02) 2.74
Siddharth 1985 0.00 (0.00, 0.02) 2.74
DeBeer 1965 0.00 (0.00, 0.01) 2.81
Williams 1934 0.00 (0.00, 0.00) 2.86
Ming-Tzu 1937 0.00 (0.00, 0.01) 2.79
del Sol 2011 0.01 (0.00, 0.05) 2.73
Darji 2015 0.00 (0.00, 0.01) 2.78
Chung 1976 0.00 (0.00, 0.08) 2.27
Lipschutz 1918 0.00 (0.00, 0.02) 2.74
Odgen 1974 0.00 (0.00, 0.05) 2.51
Bloda 1982 0.00 (0.00, 0.02) 2.71
Boraiah 2009 0.00 (0.00, 0.12) 2.09
Liu 2001 0.00 (0.00, 0.03) 2.61
Luo 1997 0.00 (0.00, 0.03) 2.61
Overall 0.00 (0.00, 0.02) 100.00
Q=774.27, p=0.00, I2=95%
0
0.05
0.1
Prev

From external iliac artery

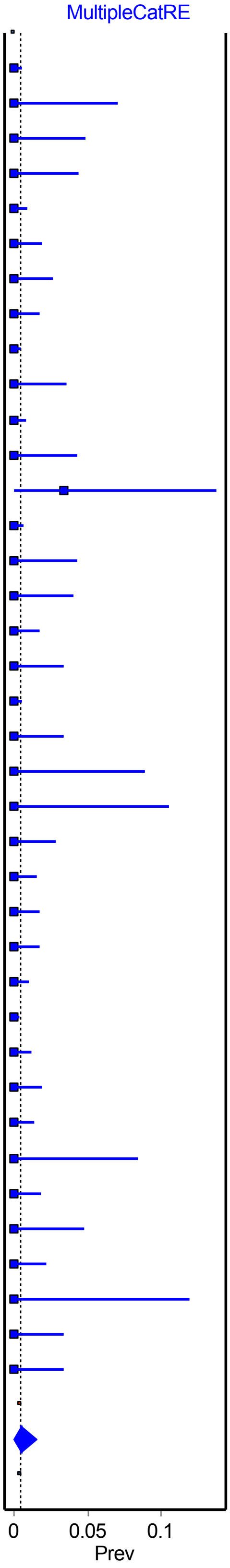

Study	Prev (95% CI)	% Weight
Al-Talalwah 2015	0.00 (0.00, 0.01)	2.85
Gautier 2000	0.00 (0.00, 0.07)	2.36
Kalhor 2012	0.00 (0.00, 0.05)	2.50
Manjappa 2014	0.00 (0.00, 0.04)	2.54
Massoud 1997	0.00 (0.00, 0.01)	2.81
Nasr 2014	0.00 (0.00, 0.02)	2.73
Prakash 2010	0.00 (0.00, 0.03)	2.66
Tanyeli 2006	0.00 (0.00, 0.02)	2.74
Vazquez 2007	0.00 (0.00, 0.00)	2.86
Dixit 2001	0.00 (0.00, 0.04)	2.60
Dixit 2011	0.00 (0.00, 0.01)	2.83
Peera 2013	0.00 (0.00, 0.04)	2.54
Clarke 1993	0.03 (0.00, 0.14)	2.45
Keen 1961	0.00 (0.00, 0.01)	2.84
Shiny Vinila 2013	0.00 (0.00, 0.04)	2.54
Lalovic 2013	0.00 (0.00, 0.04)	2.56
Goscicka 1990	0.00 (0.00, 0.02)	2.74
Marcade 1978	0.00 (0.00, 0.03)	2.61
Emura 1989	0.00 (0.00, 0.01)	2.85
Munteanu 1998	0.00 (0.00, 0.03)	2.61
O'Hara 1983	0.00 (0.00, 0.09)	2.25
Zlotorowicz 2013	0.00 (0.00, 0.11)	2.16
Anwer 2013	0.00 (0.00, 0.03)	2.65
Boonkham 1987	0.00 (0.00, 0.02)	2.76
Gremigni 1968	0.00 (0.00, 0.02)	2.74
Siddharth 1985	0.00 (0.00, 0.02)	2.74
DeBeer 1965	0.00 (0.00, 0.01)	2.81
Williams 1934	0.00 (0.00, 0.00)	2.86
Ming-Tzu 1937	0.00 (0.00, 0.01)	2.79
del Sol 2011	0.00 (0.00, 0.02)	2.73
Darji 2015	0.00 (0.00, 0.01)	2.78
Chung 1976	0.00 (0.00, 0.08)	2.27
Lipschutz 1918	0.00 (0.00, 0.02)	2.74
Odgen 1974	0.00 (0.00, 0.05)	2.51
Bloda 1982	0.00 (0.00, 0.02)	2.71
Boraiah 2009	0.00 (0.00, 0.12)	2.09
Liu 2001	0.00 (0.00, 0.03)	2.61
Luo 1997	0.00 (0.00, 0.03)	2.61
Overall	0.00 (0.00, 0.02)	100.00

Q=774.27, p=0.00, I2=95%

Aplasia

Study	Prev (95% CI)	% Weight
Al-Talalwah 2015	0.01 (0.00, 0.02)	2.85
Gautier 2000	0.00 (0.00, 0.07)	2.36
Kalhor 2012	0.00 (0.00, 0.05)	2.50
Manjappa 2014	0.00 (0.00, 0.04)	2.54
Massoud 1997	0.03 (0.01, 0.06)	2.81
Nasr 2014	0.00 (0.00, 0.02)	2.73
Prakash 2010	0.00 (0.00, 0.03)	2.66
Tanyeli 2006	0.00 (0.00, 0.02)	2.74
Vazquez 2007	0.00 (0.00, 0.00)	2.86
Dixit 2001	0.00 (0.00, 0.04)	2.60
Dixit 2011	0.00 (0.00, 0.01)	2.83
Peera 2013	0.00 (0.00, 0.04)	2.54
Clarke 1993	0.00 (0.00, 0.06)	2.45
Keen 1961	0.00 (0.00, 0.01)	2.84
Shiny Vinila 2013	0.00 (0.00, 0.04)	2.54
Lalovic 2013	0.05 (0.00, 0.14)	2.56
Goscicka 1990	0.00 (0.00, 0.02)	2.74
Marcade 1978	0.00 (0.00, 0.03)	2.61
Emura 1989	0.00 (0.00, 0.01)	2.85
Munteanu 1998	0.00 (0.00, 0.03)	2.61
O'Hara 1983	0.00 (0.00, 0.09)	2.25
Zlotorowicz 2013	0.00 (0.00, 0.11)	2.16
Anwer 2013	0.00 (0.00, 0.03)	2.65
Boonkham 1987	0.00 (0.00, 0.02)	2.76
Gremigni 1968	0.00 (0.00, 0.02)	2.74
Siddharth 1985	0.00 (0.00, 0.02)	2.74
DeBeer 1965	0.00 (0.00, 0.01)	2.81
Williams 1934	0.00 (0.00, 0.00)	2.86
Ming-Tzu 1937	0.00 (0.00, 0.01)	2.79
del Sol 2011	0.00 (0.00, 0.02)	2.73
Darji 2015	0.00 (0.00, 0.01)	2.78
Chung 1976	0.00 (0.00, 0.08)	2.27
Lipschutz 1918	0.00 (0.00, 0.02)	2.74
Odgen 1974	0.00 (0.00, 0.05)	2.51
Bloda 1982	0.01 (0.00, 0.05)	2.71
Boraiah 2009	0.00 (0.00, 0.12)	2.09
Liu 2001	0.00 (0.00, 0.03)	2.61
Luo 1997	0.00 (0.00, 0.03)	2.61
Overall	0.01 (0.00, 0.02)	100.00

Q=774.27, p=0.00, I2=95%

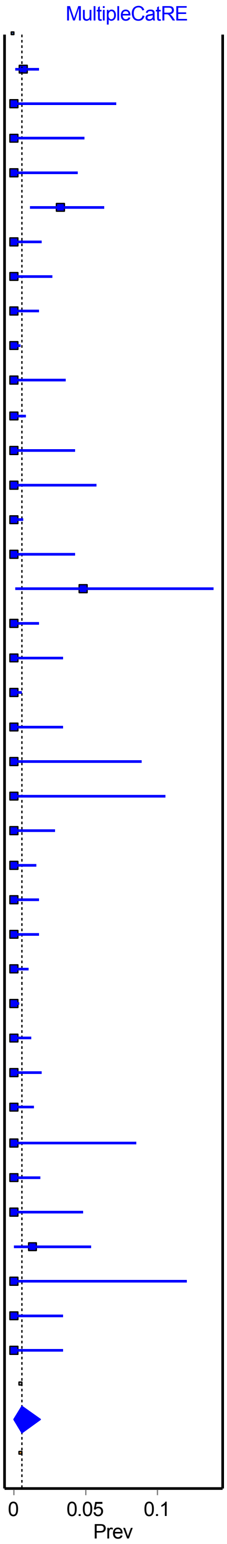

Supplement: Supplemental Information 2 [file peerj-04-1726-s003.pdf]
